# Non-Coding Structural Variants in Regulatory Regions and High Polygenic Burden May Jointly Contribute to Early-Onset Familial Hashimoto’s Thyroiditis

**DOI:** 10.3390/genes17101303

**Published:** 2026-10-20

**Authors:** Vesna Boraska Perica, Ana Barić Žižić, Sanda Sladić, Marko Vuletić, Ivana Listeš, Dean Kaličanin, Vanna Žnidar, Vedrana Čikeš Čulić, Vesela Torlak Lovrić, Ante Punda, Veselin Škrabić, Maja Barbalić

**Affiliations:** 1 Department of Medical Biology, School of Medicine, University of Split, 21000 Split, Croatia; 2 Department of Nuclear Medicine, University Hospital of Split, 21000 Split, Croatia; 3 Department of Medical Chemistry and Biochemistry, School of Medicine, University of Split, 21000 Split, Croatia; 4 School of Medicine, University of Mostar, 88000 Mostar, Bosnia and Herzegovina; 5 Department of Biology, Faculty of Science, University of Split, 21000 Split, Croatia

**Keywords:** autoimmunity, long-read sequencing, whole-exome sequencing, polygenic risk score, Hashimoto’s thyroiditis

## Abstract

Background/Objectives: Multigenerational families with unusually early disease onset provide a unique opportunity to investigate the genetic architecture underlying the missing heritability of complex diseases. Here, we investigated the genetic basis of apparent autosomal dominant early-onset Hashimoto’s thyroiditis (HT) in a multigenerational pedigree using whole-exome sequencing (WES) in 9 individuals and PacBio HiFi long-read whole-genome sequencing (LR-WGS) in four family members spanning three generations. Methods: WES identified a rare disease-segregating *TPH2* missense variant, while LR-WGS identified approximately 2000 structural variants (SVs) shared by the three sequenced affected relatives and absent from the unaffected father. Results: Affected-lineage-shared SVs were significantly enriched within established hypothyroidism GWAS loci (447 observed vs. 292.6 expected overlaps; *p* = 6.99 × 10^−4^), with prominent clusters at the *NFATC1-ATP9B* and *PDCD6-AHRR* loci. Integrative analyses incorporating regulatory annotation, GWAS overlap, and exploratory proteomic profiling highlighted candidate regulatory SVs affecting enhancers and long non-coding RNAs within the *TPH2/TRHDE*, *TPO/LALTOP*, and *TSHR* loci. Exploratory proteomic profiling was consistent with involvement of the *NFATC1-ATP9B* and *PDCD6-AHRR* loci and identified *AXIN1* as an additional candidate locus. Parallel polygenic risk score (PRS) analysis showed that the three affected sequenced family members ranked above the 95th percentile of the HT PRS distribution. Conclusions: Together, these findings are consistent with a multi-hit genetic model in which elevated polygenic burden and multiple regulatory structural variants may jointly contribute to early-onset familial HT. The observed enrichment of affected-lineage-shared SVs within established hypothyroidism GWAS loci further suggests that regulatory structural variation may contribute to previously unexplained non-coding genetic susceptibility.

## 1. Introduction

Hashimoto’s thyroiditis (HT) is a chronic autoimmune thyroid disorder characterized by lymphocytic infiltration of the thyroid gland and the gradual destruction of thyrocytes, leading to subclinical or overt hypothyroidism in most cases. It is widely recognized as one of the most common autoimmune diseases globally and the leading cause of hypothyroidism in iodine-sufficient populations [1]. The disease typically presents with elevated serum autoantibodies against thyroid peroxidase (TPO) and/or thyroglobulin (Tg), and histopathologically with dense lymphocytic infiltration of thyroid tissue [2]. HT exhibits a strong sex bias, occurring several-fold more frequently in women than in men [3].

Familial and twin studies have demonstrated a substantial heritable component to thyroid autoimmunity [4,5], with HT generally regarded as a polygenic and multifactorial disorder [6]. Genome-wide association studies (GWASs) have so far identified well over a hundred common variants associated with HT and hypothyroidism, each individually conferring small effects on disease risk. Monogenic form has also been described in a multigenerational family with early-onset HT and autosomal dominant inheritance, in whom whole-exome sequencing (WES) identified a disease-segregating splice site variant in the *TG* gene [7]. Such a monogenic explanation, however, accounts for only a small fraction of familial clustering, and a considerable proportion of HT heritability remains unexplained.

A central limitation of current genetic studies is that they have predominantly focused on the analysis of single-nucleotide polymorphisms (SNPs) or short-read exome data. Structural variants (SVs), including deletions, insertions, inversions, and complex rearrangements, are systematically under-detected by these approaches, despite increasing evidence that they contribute substantially to phenotypic variation and disease risk through disruption of regulatory architecture, including enhancers, long non-coding RNAs (lncRNAs), and three-dimensional chromatin interactions [8,9]. Long-read whole-genome sequencing (LR-WGS) now enables comprehensive characterization of SVs across the entire genome, offering an opportunity to investigate a class of variation that has largely been invisible in HT genetics. In parallel, polygenic risk scores (PRSs) derived from large-scale GWASs now allow quantification of an individual’s cumulative common-variant burden, providing a framework to assess whether familial disease clustering can be explained by an unusually high concentration of common risk alleles, comparable to the burden conferred by rare monogenic mutations [10].

**Figure 1:**
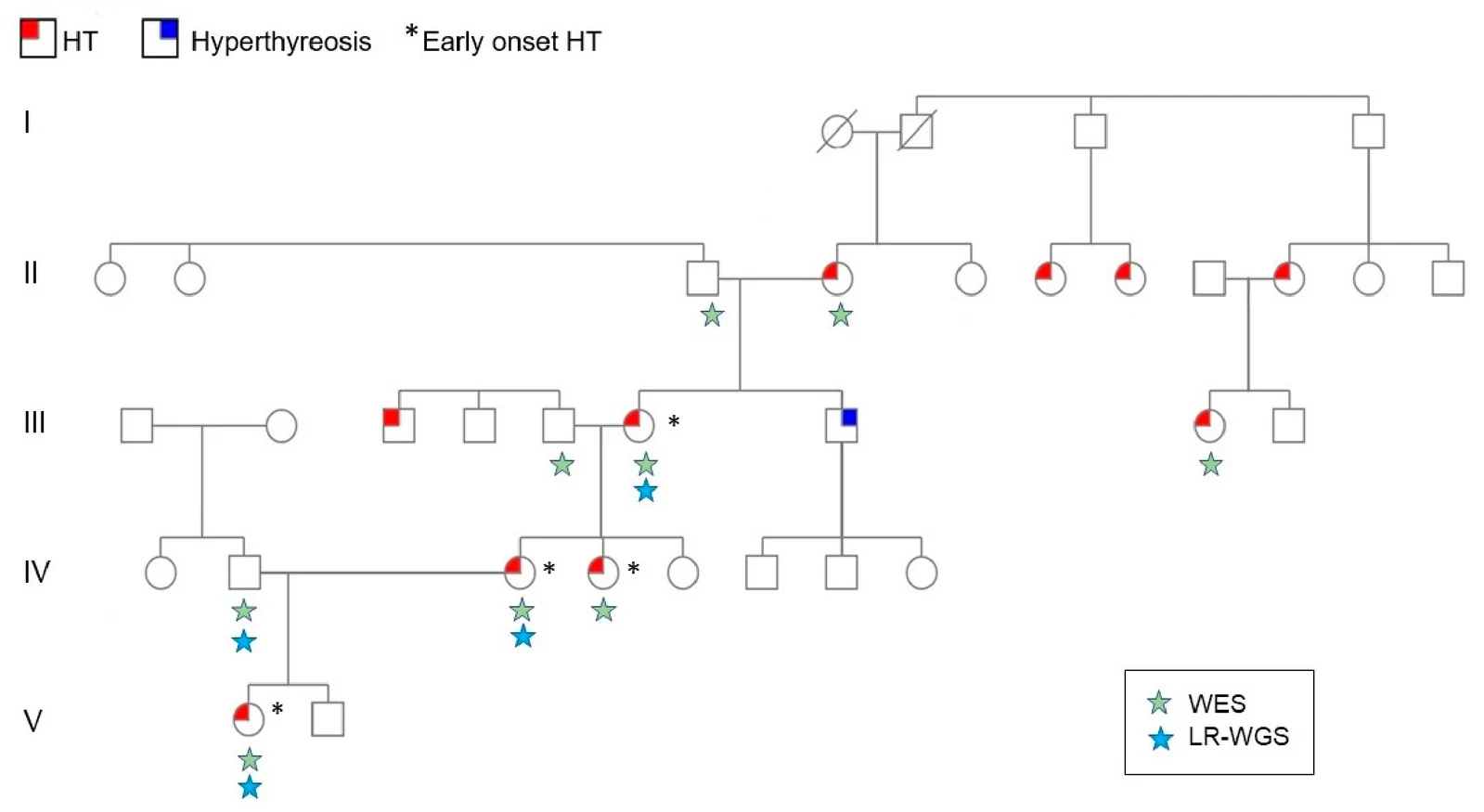
Pedigree chart of a family with multiple cases of early onset Hashimoto’s thyroiditis.

Against this background, multigenerational families with unusually early disease onset represent informative natural experiments for dissecting the relative contributions of rare and common variations. In this study, we investigated such a pedigree comprising 15 individuals across four generations, with six members diagnosed with HT, four of whom presented with childhood-onset disease (5–12 years of age), overt hypothyroidism, and markedly elevated anti-thyroid autoantibodies. Disease occurrence showed apparent vertical, dominant-like transmission, with affected individuals in four generations; given the high prevalence and female predominance of HT, incomplete penetrance in male relatives is likely (Figure 1).

We applied a three-tiered genomic strategy designed to discriminate between three competing hypotheses for the observed familial clustering: (i) a single high-impact rare variant consistent with classical Mendelian inheritance, (ii) an exceptional polygenic burden of common risk alleles, or (iii) a multi-hit architecture in which both rare structural variation and high polygenic load jointly lower the threshold for disease manifestation. We first performed WES across nine informative family members to identify rare protein-altering variants segregating with disease. Because WES did not reveal a clearly compelling high-impact coding variant, we next applied PacBio HiFi LR-WGS across three generations to interrogate structural variation that may have been missed by short-read approaches. Finally, we calculated PRS for sequenced family members and compared them to HT cases and controls from a population-based CRO-HT biobank [11]. We also performed proteomic and metabolomic profiling of affected family members and controls to search for potential functional correlates of the genetic findings. Together, these complementary approaches were designed to clarify whether the marked clustering of HT in this pedigree reflects a monogenic cause, a polygenic mechanism, or, as our findings suggest, their combined contribution.

## 2. Materials and Methods

### 2.1. Pedigree Description, Clinical Assessment, and Sample Collection

We comprehensively characterized 15 members of a multigenerational family spanning four generations (Figure 1). Of these, six were diagnosed with HT, four of whom presented with childhood-onset disease (5–12 years of age), overt hypothyroidism, and markedly elevated anti-thyroid autoantibody titers. The distribution of affected and unaffected individuals across consecutive generations, including transmission through both maternal and informative non-maternal branches of the pedigree where applicable, suggested apparent dominant-like transmission with incomplete penetrance. An autosomal dominant model was therefore used as an operational filter for subsequent WES and LR-WGS analyses.

Clinical and phenotypic assessments were conducted according to established guidelines, which encompassed a comprehensive medical history, physical examination, thyroid ultrasound performed by a nuclear medicine specialist, a laboratory panel, and a structured questionnaire [12]. A specialized medical team comprising nuclear medicine specialists, nurses, and support staff performed recruitment and clinical evaluations at a local medical clinic on the island of Murter, Croatia, using portable high-resolution ultrasound and specialized cooling units for sample transport. Patients were enrolled in the HT group based on a known history of HT, stable levothyroxine (LT4) therapy, past diagnostic confirmation and on-site sonographic evidence of diffuse thyroid disease (heterogeneous and/or hypoechoic parenchyma). All HT patients from this family had an established history of HT and were receiving LT4 treatment, meaning further clinical subclassification was not applicable. Comprehensive data on thyroid hormones/antibodies and other relevant clinical information for all enrolled family members are presented in Table 1. Alongside physical examinations, a wide range of phenotypes were collected via structured questionnaires or directly measured during the participants’ enrollment.

Following on-site collection, venous blood samples were transported to the Clinical Department of Nuclear Medicine at the University Hospital of Split, where samples were processed and aliquoted. Serum concentrations of thyroid-stimulating hormone (TSH), free thyroxine (FT4), free triiodothyronine (FT3), and thyroid-specific autoantibodies (TgAb, TPOAb) were measured using chemiluminescence immunoassays on the LIAISON platform (DiaSorin, Saluggia, Italy). The laboratory reference intervals were as follows: TSH [0.3–3.6] mIU/L, FT4 [10.3–22.8] pmol/L, FT3 [3.13–6.47] pmol/L, TgAb [5–100] IU/mL, and TPOAb [1–16] IU/mL.

### 2.2. Whole-Exome Sequencing

Nine of the fifteen clinically characterized family members were selected for WES based on their clinical status, position within the pedigree and expected informativeness for segregation analysis. These included six affected individuals representing multiple generations and branches of the family, as well as three unaffected relatives who were spouses of affected individuals and fathers of affected offspring. This selection was intended to facilitate the identification of rare variants shared among affected relatives and assessment of their inheritance within the pedigree while taking into account the available sequencing resources (Figure 1, Table 1). Exome capture and high-throughput sequencing were performed by Celemics, Inc. (Seoul, Republic of Korea). The process included genomic DNA quality control (gDNA QC) and WES library preparation, followed by sequencing on the Illumina platform with a target depth of >100x coverage per sample.

Raw FASTQ files were processed using the Genomize SEQ Platform (Genomize, Istanbul, Turkey), which provides automated workflows for read alignment to the human reference genome (GRCh37/hg19) and variant calling via the Genome Analysis Toolkit (GATK) [13]. A multi-step filtering strategy was applied: first, a familial segregation filter retained only variants heterozygous in all affected individuals and homozygous wild-types in all unaffected relatives, consistent with the hypothesized autosomal dominant inheritance pattern; second, variants with minor allele frequency (MAF) > 0.01 in gnomAD v2.1.1 [14] were excluded; and finally, the remaining variants were inspected for predicted functional impact, with a focus on rare protein-altering coding variants. Full details of the annotation resources and filtering strategy are provided in the Supplemental Materials. This strategy prioritized a single rare missense variant in the *TPH2* (Tryptophan Hydroxylase 2) gene.

To explore the possible functional impact of this prioritized variant, we quantified plasma levels of 5-hydroxytryptophan (5-HTP), a product of TPH enzyme activity, which catalyzes the conversion of tryptophan to 5-HTP. A complementary motivation for prioritizing this variant was its close genomic proximity to *TRHDE* (Thyrotropin-Releasing Hormone-Degrading Enzyme), a regulator of the hypothalamic–pituitary–thyroid (HPT) axis, leading us to hypothesize that the missense variant might tag a co-segregating structural variant on the same haplotype.

### 2.3. 5-HTP Quantification and Statistical Analysis

Plasma levels of 5-HTP were measured using a competitive inhibition enzyme-linked immunosorbent assay (ELISA; ELK Biotechnology, Wuhan, China) in all sequenced affected family members (mutation carriers) and 19 controls selected from previously recruited participants within the CRO-HT biobank [11]. The assay was performed in duplicate, and concentrations (ng/mL) were quantified by comparison against a standard calibration curve. Demographic and clinical characteristics of the affected family members and the 19 controls are presented in Supplementary Table S1. Both cohorts followed an identical long-term preservation protocol, whereby all serum samples were aliquoted and stored at −80 °C. Regarding pre-analytical processing, control samples obtained from the CRO-HT biobank were centrifuged within 1–3 h of collection. Family samples were collected on site, immediately placed on ice to arrest ex vivo metabolic activity, transported to the laboratory, and centrifuged within 8 h.

Because exploratory inspection of 5-HTP values revealed a markedly right-skewed distribution (with the standard deviation in controls exceeding the mean), differences between affected family members and controls were assessed using a non-parametric Mann–Whitney U test as the primary analysis. We note that, given the small number of affected carriers (*n* = 6) and controls (*n* = 19), the analysis is underpowered to detect modest differences. Furthermore, plasma 5-HTP primarily reflects peripheral TPH1 rather than neuronal TPH2 activity, which further limits its interpretation as a direct biomarker of TPH2 function. Statistical analyses were performed using SPSS version 20 (SPSS Inc., Chicago, IL, USA); significance was defined as *p* < 0.05.

### 2.4. Long-Read Whole-Genome Sequencing

To further characterize the genomic region surrounding the candidate *TPH2-TRHDE* region and to evaluate structural variation on a genome-wide scale, LR-WGS was performed on four family members spanning three generations: the affected proband (daughter), her affected mother and grandmother, and her unaffected father (Figure 1). Sequencing was carried out using PacBio HiFi technology on the Sequel II/IIe platform (Novogene GmbH, Munich, Germany). Circular Consensus Sequencing (CCS) generated highly accurate HiFi reads (QV ≥ 20), which were aligned to GRCh38 using pbmm2. Technical details on library construction are provided in the Supplemental Materials.

Aligned BAM files were analysed using the varvis^®^ Platform (Limbus Medical Technologies GmbH, Rostock, Germany). Given the objectives of this study, downstream analyses focused primarily on the detection and interpretation of SVs.

### 2.5. Structural Variant Filtering

For segregation analysis, SVs detected in the affected proband were used as reference positions. An SV was retained if it was called, with the same SV type, in the proband, her mother and her grandmother, and was not called in the father within a ±100 bp window around its breakpoints, to account for breakpoint uncertainty in long-read SV calling. This approach isolated SVs inherited through the affected lineage (grandmother → mother → daughter), hereafter referred to as affected-lineage-shared SVs. Because LR-WGS was performed only in three affected relatives in a direct line of descent and one unaffected individual, sharing of an SV does not by itself establish co-segregation with disease across the pedigree.

### 2.6. Prioritization Strategy 1: Clinical Classification, Pathogenicity Scoring, and Disease-Targeted Gene Filtering

Annotation of affected-segregating SVs was performed using the web version of the AnnotSV tool [15]. For downstream interpretation, we focused on SVs assigned an American College of Medical Genetics and Genomics (ACMG) classification score ≥3, corresponding to variants of uncertain significance, variants likely pathogenic, and pathogenic variants [16,17]. Further prioritization focused on SVs located in established HT-associated genes (such as *TPO* and *TSHR*). Prioritization was based on the existence of SVs intersecting regulatory elements. Cis-regulatory annotation was performed via ENCODE SCREEN https://screen.encodeproject.org/ (accessed on the 11 June 2026), ChIA-PET chromatin interaction data was also inspected through the ENCODE portal, and eQTL lookup was searched via the GTEx eQTL Calculator v10 https://gtexportal.org/ (accessed on the 11 June 2026). Technical details on AnnotSV annotation and analysis are provided in the Supplemental Materials.

### 2.7. Prioritization Strategy 2: Hypothyroidism-Associated GWAS Overlap and Enrichment Analysis

Annotation and prioritization of affected-segregating SVs were extended based on their genomic overlap with known SNPs associated with hypothyroidism retrieved from the GWAS Catalog https://www.ebi.ac.uk/gwas/ (accessed on the 16 March 2026). All coordinates were harmonized to GRCh38 prior to overlap analysis. Unique SNPs were pruned for linkage disequilibrium (LD) using LDlink https://ldlink.nih.gov/ (accessed on the 15 March 2026), yielding 607 independent hypothyroidism-associated variants.

To capture wider regulatory or haploblock regions tagged by these variants, a genomic window was constructed for each SV by extending 500 kb upstream and 500 kb downstream from its boundaries. Overlap analysis was performed by intersecting the 607 independent hypothyroidism SNPs with these SV-defined windows, revealing 447 overlaps corresponding to 209 unique SV regions and 173 unique GWAS SNPs (Supplementary Table S2). Because neighboring SV windows can overlap and a single GWAS SNP can contribute to multiple window intersections, reporting the count of unique GWAS SNPs was utilized to control for data dependence at the window level.

To assess whether the observed overlap exceeded random expectation, an empirical enrichment analysis was conducted. A background pool was generated from the entire GWAS Catalog by retaining unique SNPs and applying LD pruning with PLINK [18], yielding 297,191 independent variants for the CEU reference population. From this background, 607 SNPs (matching the size of the hypothyroidism-associated set) were randomly sampled over 10,000 permutations. For each iteration, the number of overlaps with our affected-overlapping SV regions was computed using identical criteria. Crucially, the genomic architecture and overlap structure of the SV windows were kept strictly fixed across all permutations, thereby inherently preserving and accounting for the topological dependence of the windows within the empirical null distribution. Empirical *p* values were calculated as the proportion of permutations yielding a number of overlaps equal to or greater than the observed value.

### 2.8. Prioritization Strategy 3: Proteomics-Guided Cross-Cohort Integration

To extend genomic prioritization beyond structural annotation and GWAS overlap, we integrated exploratory plasma proteomic data from affected family members (Supplemental Note S1) with protein-level data from an independent cross-sectional study of HT from the CRO-HT biobank [19], which measured 92 inflammation-associated proteins using the Olink Target 96 Inflammation panel. Affected family members (*n* = 7) and euthyroid HT patients on LT4 replacement therapy (*n* = 150) were independently compared against population-based controls (*n* = 209), followed by a comparative analysis of the summary results. This analysis was performed for two complementary purposes: (a) we tested whether the proteins significantly associated with HT that overlapped across both analyses map to genes prioritized by our two prioritization strategies (per above), which may provide independent functional evidence that these loci are biologically active; (b) second, for proteins showing a family-specific signal (significant in families but non-significant in the population cohort), we performed targeted genomic (SV) follow-up.

### 2.9. Metabolic Profiling

Metabolomic profiling using the Nightingale Health platform (250 metabolic measures) was performed in the same participants and is concisely reported here, as it did not provide mechanistic insight independent of the genomic findings. Full findings are reported in Supplemental Note S2.

### 2.10. Polygenic Risk Score Calculation

PRSs were calculated for all four family members with LR-WGS data and, in parallel, for HT cases and controls from the CRO-HT biobank using imputed genotype data (Supplemental Materials). The PRS used risk variants, effect alleles, and effect sizes from the latest multi-ancestry GWAS on HT [20], and was computed as PRS = Σ (β_i_ × G_i_), where β_i_ is the effect size of variant i, and G_i_ ∈ {0, 1, 2} is the number of effect alleles carried by the individual. Of the 155 HT-associated variants reported in the original study, 134 were directly available in our genotype data. For 13 of the missing variants, suitable proxies (R^2^ > 0.8) were identified using LDproxy, yielding a final PRS based on 147 variants (134 original and 13 proxies). The remaining eight variants were excluded because no proxy met the threshold (*n* = 2), the variant was absent from the LDproxy database (*n* = 1), the identified proxies were absent from our genotype data (*n* = 2), or LDproxy returned proxies for a different variant in the same genomic position (*n* = 3) (Supplemental Materials).

## 3. Results

### 3.1. WES Analysis

Application of the familial segregation filter yielded 359 SNVs that were heterozygous in all affected individuals and absent in unaffected family members. Subsequent filtering for rare variants (MAF < 0.01) reduced this set to 26 variants (Supplementary Figure S1 and Table S3). Of these, 25 were located in non-coding regions without clear evidence of functional relevance, leaving a single protein-altering variant: a heterozygous missense variant in *TPH2* (rs17110563; c.616C > T; p.Pro206Ser). The variant is rare in population databases (gnomAD genome frequency ~0.001) and segregated with disease in all six affected family members. Its close genomic proximity to *TRHDE*, a regulator of the HPT axis, motivated further functional and structural investigations.

### 3.2. 5-HTP Measurement

To assess the potential functional impact of the identified SNV within *TPH2*, we measured plasma 5-HTP, the direct product of TPH enzymatic activity. Affected carrier family members had lower mean 5-HTP levels (47.01 ± 41.58 ng/mL) than controls (98.39 ± 140.71 ng/mL); however, the difference was not statistically significant (Mann–Whitney U, *p* = 0.36; sensitivity analysis: logistic regression on log-transformed values, OR 0.99, 95% CI 0.97–1.01, *p* = 0.36). Given the small sample size and the fact that plasma 5-HTP predominantly reflects peripheral TPH1 rather than neuronal TPH2 activity, these data neither support nor refute a functional effect of the variant, but they do not provide positive evidence for a major functional role of the *TPH2* variant in disease pathogenesis. However, the functional impact of TPH2 p.Pro206Ser has previously been evaluated in vitro by Cichon et al., who expressed wild-type and mutant TPH2 in *Escherichia coli* and human embryonic kidney (HEK293) cells and showed that, although the mutant retained catalytic activity (Vmax) similar to the wild-type enzyme, it had reduced solubility and thermal stability, with a 3–6-fold higher rate of inactivation at 37–55 °C [21].

### 3.3. TPH2/TRHDE Locus

Within the genomic region surrounding the *TPH2* missense variant identified by WES, LR-WGS revealed a 503 bp heterozygous deletion within the *TPH2* gene, present in the three affected family members and absent in the unaffected father, thereby mirroring the segregation pattern of the WES variant (Table 2). The deletion has a population frequency of 0.0795 in the CoLoRS database https://colorsdb.org/ (accessed on the 11 June 2026) and encompasses approximately 85% of the candidate cis-regulatory element cCRE EH38E1624299 [22], which, according to ENCODE data, functions as a topological anchor mediating 3D chromatin looping between the *TPH2* locus and the adjacent region [22]. A variant within the deleted region (rs1352251) is associated with reduced *TRHDE* expression in spinal cord (*p* = 4.27 × 10^−3^, Supplemental Figure S2) and coronary artery (*p* = 1.12 × 10^−2^) tissues, as identified using the GTEx eQTL Calculator [23]. We emphasize that these eQTL signals derive from non-thyroid, non-HPT-relevant tissues, which limits direct mechanistic inference. Further information is provided in the Supplemental Materials.

### 3.4. Prioritization Strategy 1: Clinical Classification, Pathogenicity Scoring, and Disease-Targeted Gene Filtering

LR-WGS in three affected family members and the unaffected father identified 2000 SVs present in all three affected individuals across three generations and absent in the unaffected father (affected-lineage-shared SVs) (Supplementary Table S4).

Annotation of 2000 affected-segregating SVs using AnnotSV yielded 124 variants classified as variants of uncertain significance (Supplementary Figure S1 and Table S5), with no variants categorized as likely pathogenic or pathogenic. This scarcity of individually pathogenic SVs argues against a classical single-variant explanation for the family’s phenotype and motivated the subsequent integrative analyses. Prioritized SVs are detailed below (*TPO*/*LALTOP* and *TSHR*).

### 3.5. TPO/LALTOP Locus

AnnotSV-based prioritization highlighted two deletions in the *TPO/LALTOP* region (an established HT-associated region) for closer inspection (Table 2 and Table S5).

A 72 bp deletion overlaps the intronic region of *TPO* and the lncRNA *LALTOP*, which is predominantly expressed in thyroid tissue (CoLoRS frequency 0.267344). The GTEx eQTL Calculator identified significant thyroid-specific cis-eQTL signals affecting *LALTOP* (*ENSG00000228613*) and an additional lncRNA *ENSG00000283766*. Variants located within or adjacent to the deleted segment (e.g., rs77496745, rs28991270) were associated with reduced expression of both lncRNAs in thyroid tissue (Supplemental Figure S2). In contrast, no strong thyroid-specific cis-eQTL signal was detected for the *TPO* gene itself, despite its high thyroid expression.

A second 116 bp deletion overlaps both the *TPO* intron and the *LALTOP* transcript (Table 2), and partially overlaps the distal enhancer cCRE EH38E3320015 (covering 31%/77 bp of this element). This variant was not present in the CoLoRS database, indicating its rare frequency. Further information is provided in the Supplemental Materials.

### 3.6. TSHR Locus

A 38 bp deletion was identified approximately 316 kb downstream of *TSHR* (Table 2) (CoLoRS frequency 0.2516). It does not overlap any annotated regulatory elements; however, a nearby cCRE (EH38E4111417), located ~746 bp from the variant, shows chromatin interactions with the *TSHR* locus based on Hi-C data. In the absence of direct regulatory overlap or supporting functional evidence, this SV is reported as an exploratory candidate, and its biological relevance remains uncertain (Table 2). Further information is provided in the Supplemental Materials.

### 3.7. Prioritization Strategy 2: Enrichment of Affected-Segregating SVs at Hypothyroidism GWAS Loci

To test whether the 2000 affected-segregating SVs preferentially localized to known disease loci, we evaluated their overlap with 607 LD-pruned hypothyroidism-associated GWAS SNPs (using ±500 kb windows around each SV) and compared the observed overlap against 10,000 permutations of size-matched random SNP sets drawn from the full GWAS Catalog.

The observed number of overlaps (*n* = 447) substantially exceeded the null expectation (mean = 292.6), corresponding to an empirical *p* = 6.99 × 10^−4^. Similarly, the number of unique GWAS SNPs falling within SV windows (*n* = 173) exceeded the permutation-based expectation (mean = 134.9; empirical *p* = 2.99 × 10^−4^), indicating a significant enrichment of hypothyroidism GWAS signals within SV regions. In contrast, the number of unique SV regions harbouring at least one hypothyroidism-associated variant (*n* = 209) did not differ significantly from expectation (mean = 261.2; empirical *p* = 0.96), suggesting that while GWAS signals are enriched within SV regions, they tend to cluster within a subset of regions rather than being broadly distributed across all regions, with the strongest clustering observed in the *NFATC1-ATP9B* and *PDCD6-AHRR* regions (Supplementary Table S4). This genome-wide spatial association represents the principal molecular finding of the study and motivated the subsequent locus-specific characterization of SVs in these loci.

### 3.8. NFATC1-ATP9B Locus

Within the *NFATC1-ATP9B* gene region, we found a particularly striking example of SV clustering in a known hypothyroidism-associated region with 23 SVs overlapping a region containing 3 GWAS hits (Table 2 and Table S4) and multiple enhancer elements.

Within this cluster, we pinpointed a 49 bp insertion located within the enhancer EH38E3278273 (CoLoRS frequency 0.045), which functions as a promoter in B cells and also acts as an eQTL for a thyroid-expressed lncRNA *ENSG00000267287* (rs1660155, positioned 19 bp upstream of the SV, *p* = 4.05 × 10^−8^; rs1245859817, *p* = 1.4 × 10^−4^; Supplemental Figure S2). Furthermore, a 1583 bp insertion lies within enhancer EH38E4188896 (CoLoRS frequency 0.017), with associated eQTL signals at rs368752539 (~200 bp upstream of the insertion) for *ENSG00000279077* in thyroid (*p* = 1.1 × 10^−5^; Supplemental Figure S2). Three additional SVs (263 bp and 184 bp deletions and a 75 bp insertion) overlap four distal enhancers (EH38E3278311, EH38E3278313, EH38E3278314, EH38E3278315) that show chromatin interactions with the *NFATC1* gene and with various immune cell types, including B, T, and NK cells. The two deletions were absent from CoLoRS, while the 75 bp insertion had a reported frequency of 0.0332866. Further information is provided in the Supplemental Materials.

### 3.9. PDCD6-AHRR Locus

A third clustering of 10 SVs was identified within the *PDCD6-AHRR* locus, overlapping four distal enhancer elements supported by multiple eQTL signals and chromatin interaction data (Table 2 and Table S4). A strong candidate within this cluster is a 40 bp insertion (absent from CoLoRS) that overlaps enhancer EH38E2351870, which shows multiple eQTL associations involving eleven genes. The variant rs7379162, located 5 bp upstream of the insertion and within the same enhancer region, is strongly associated with *PDCD6-DT* (PDCD6 Divergent Transcript, lncRNA) expression in thyroid tissue (*p* = 1.83 × 10^−25^; Supplemental Figure S2). Further information is provided in the Supplemental Materials.

The finding of clusters of SVs in GWAS signals prompted us to explore the whole set of 2000 affected-segregating SVs and search for additional clusters of SVs in thyroid-related genes. The most compelling result was a finding of 24 SVs in the *PTPRN2* locus, already associated with TPOAb antibodies in our population [24]. Several SVs in this locus overlapped regulatory elements, suggesting potential functional relevance. Detailed analysis of this cluster is provided in the Supplemental Materials.

### 3.10. Prioritization Strategy 3: Proteomics-Guided Cross-Cohort Integration

(a) Proteomic data were consistent with involvement of the *NFATC1-ATP9B* and *PDCD6–AHRR* loci

Levels of eleven proteins were significantly different in affected family members versus population-based controls (Supplementary Table SN1). Reduced proteins included SIRT2, CXCL1, CXCL5, CXCL6, IL-7, AXIN1, ST1A1 and STAMBP, whereas TNFRSF9, EN-RAGE and IL-12B were increased. Of these, nine overlapped with results from LT4 versus control comparison (except EN-RAGE and AXIN1). Based on the literature, ten of the eleven proteins (all except AXIN1) can be linked to pathways associated with the two SV cluster loci (*NFATC1-ATP9B* and *PDCD6–AHRR*). Six proteins link to the *NFATC1-ATP9B* locus (CXCL1, CXCL5, CXCL6, IL-7, TNFRSF9, IL-12B) and four to the *PDCD6–AHRR* locus (SIRT2, ST1A1, STAMBP, EN-RAGE) (detailly discussed in Supplemental Note S1).

(b) Cross-cohort integration identifies *AXIN1* as an additional candidate locus

### 3.11. AXIN1 Locus

In affected family members, *AXIN1* displayed a direction of effect opposite to that observed in the LT4-treated HT patients; thus, this family-specific signal motivated a targeted SV search in the *AXIN1* region (chr16p13.3). Seven SVs were identified within *AXIN1* genomic region; none mapped to the gene body (Table 2 and Table S4). The primary candidate is a 585 bp deletion (~127 kb upstream; absent from CoLoRS), overlapping two ENCODE Candidate Activator cCREs (EH38E4133037 and EH38E4133038). A second SV (87 bp deletion, chr16:123,899–123,986; CoLoRS AF = 0.049) overlaps two proximal enhancers (EH38E4133026, EH38E4133027). The variants rs868300042 (within EH38E4133038) and rs17146023 (within EH38E4133027) are associated with *AXIN1* expression in the brain (cerebellum) (GTEx) (Figure S2).

### 3.12. Metabolic Profiling

Compared with unrelated LT4-treated HT patients, affected family members (*n* = 7) showed six nominally significant differences (*p* < 0.05) in plasma NMR metabolomic profiles. All six were in relative lipoprotein composition: higher proportions of cholesterol in VLDL particles and lower proportions of phospholipids and cholesterol esters in small HDL and IDL particles. These exploratory findings are detailed in Supplementary Note S2.

### 3.13. Polygenic Risk Score

**Figure 2:**
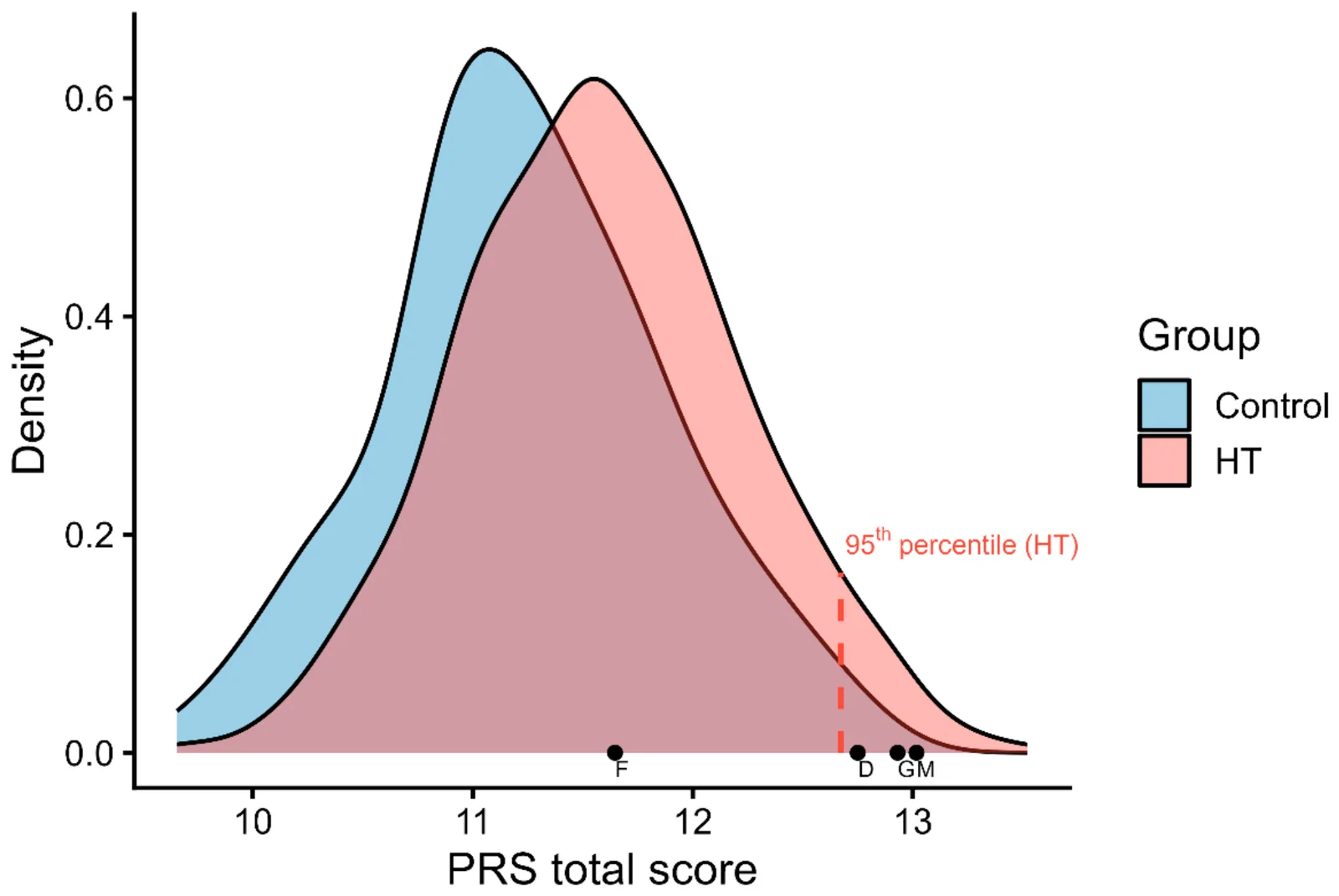
Polygenic risk score in HT cases, controls, affected family members (D—daughter, M—mother, G—grandmother), and unaffected father (F).

The three sequenced affected family members (daughter, mother, and grandmother) exhibited highly similar polygenic risk scores (mean ± SD: 12.20 ± 0.06), exceeding the mean PRS observed in both unrelated HT cases (10.90 ± 0.62) and controls (10.62 ± 0.61) from the CRO-HT biobank by approximately two standard deviations. All three sequenced affected family members ranked within the top 5% (≥95th percentile) of the HT PRS distribution. In contrast, the unaffected father exhibited a PRS value close to the population mean (Figure 2). Notably, the PRS distinguished affected family members from unrelated HT cases more clearly than it distinguished cases from controls in the broader CRO-HT biobank.

## 4. Discussion

While the striking clustering of early-onset HT in this family initially suggested a classical monogenic, autosomal dominant inheritance pattern, our findings are more consistent with a complex genomic architecture involving both common and structural genetic variation. The marked elevation of polygenic burden, the absence of a high-impact coding variant, and the genome-wide non-random distribution of segregating SVs across hypothyroidism-associated loci collectively argue against a single-gene explanation in this pedigree.

WES did not identify a clearly pathogenic coding variant. Although a rare *TPH2* missense variant segregated with disease in all six affected members, plasma 5-HTP levels did not differ significantly between carriers and controls. This result should be interpreted cautiously, as plasma 5-HTP primarily reflects peripheral TPH1 activity rather than the neuronal TPH2 isoform, and the small case–control sample limits statistical power to detect modest effects. Nevertheless, combined with the limited evidence linking serotonin biosynthesis to thyroid autoimmunity, these data provide little support for a major functional role of this variant in disease pathogenesis. Its co-segregation may instead reflect linkage to nearby regulatory variation or coincidental occurrence within a genetically enriched background.

This observation led us to perform LR-WGS to further interrogate the *TPH2* locus and systematically evaluate SVs across the genome, enabling the prioritization of loci with established roles in thyroid function and congenital hypothyroidism. The principal finding of our study emerged from the genome-wide analysis of structural variation. The 2000 SVs segregating with disease showed a significant enrichment within hypothyroidism-associated GWAS regions (empirical *p* = 6.99 × 10^−4^), with disease-associated SNPs preferentially clustering in a subset of SV-containing loci rather than being distributed uniformly across them. This non-random spatial association indicates that segregating structural variation preferentially co-localizes with established disease-susceptibility loci, supporting the hypothesis that SVs may represent an additional regulatory layer contributing to HT susceptibility in regions previously identified in SNP-based studies.

Altogether, six loci were prioritized based on complementary lines of evidence, including segregation patterns from WES (*TPH2/TRHDE*), functional annotation using AnnotSV (*TPO/LALTOP* and *TSHR*), clustering of SVs within established hypothyroidism-associated GWAS signals (the *NFATC1-ATP9B* and *PDCD6-AHRR*), and proteomic-based profiling (*AXIN1*). All prioritized SVs overlap regulatory elements, including candidate cis-regulatory elements (cCREs), enhancers, and regions supported by eQTL and chromatin interaction data (Table 2 and Table S4), suggesting a potential influence on gene expression rather than alteration of protein-coding sequences.

The *TPH2/TRHDE* locus (Table 2) is biologically interesting because the 503 bp deletion encompasses approximately 85% of enhancer EH38E1624299, which functions as a topological anchor between *TPH2* and the neighbouring *TRHDE* gene. TRHDE is a metallopeptidase responsible for the degradation of thyrotropin-releasing hormone (TRH), which is a key regulator of the HPT axis [25]. Therefore, a regulatory perturbation at this locus could potentially affect the HPT axis. We speculate that even a moderate reduction in TRHDE activity could theoretically lead to diminished TRH degradation and localized accumulation of TRH. A variant within the deleted region is associated with reduced TRHDE expression, although the available eQTL signals derive from spinal cord and coronary artery rather than from thyroid- or HPT-relevant tissues, which limits direct mechanistic inference. Dysregulation of the TRH–TSH feedback loop is known to drive persistent TSH stimulation [26,27], and chronic TSH/thyroid hormone imbalance has documented pro-inflammatory effects that can exacerbate thyroid autoimmunity [28]. Therefore, even a moderate reduction in TRHDE expression due to the heterozygous state of the deletion might lower the physiological threshold for thyroid dysregulation, a hypothesis that requires experimental testing.

The *TPO/LALTOP* locus provides a stronger functional context (Table 2). Two SVs in cis overlap regulatory elements with thyroid-specific eQTL signals affecting several lncRNAs, including *LALTOP*, which is predominantly expressed in thyroid tissue. The involvement of this locus in HT and HT-associated traits has been independently established [29,30,31], and the co-occurrence of two deletions in cis encompassing multiple regulatory elements is consistent with a cumulative regulatory effect at this locus.

The 38 bp deletion downstream of *TSHR* is more tentative (Table 2). Although it lacks direct regulatory annotations, a nearby transcription factor binding region maintains an intact Hi-C loop with the *TSHR* locus. *TSHR* encodes the receptor for TSH and is central to thyrocyte function [32]. Importantly, loss-of-function (inactivating) *TSHR* mutations cause congenital hypothyroidism through TSH resistance [33], which is conceptually relevant given the childhood onset of disease in this family across multiple generations. However, in the absence of direct regulatory overlap or functional evidence, this SV is best regarded as an exploratory candidate rather than a high-confidence finding.

Additional support for a regulatory model is provided by the *NFATC1-ATP9B* and *PDCD6-AHRR* loci, both of which harbour clusters of segregating SVs overlapping enhancer elements with established roles in immune regulation (Table 2 and Table S4). *NFATC1* encodes a transcription factor central to T-cell activation and cytokine signalling [34], with additional roles in B-cell development [35]. Moreover, genetic variation at this locus has been associated with autoimmune phenotypes and hypothyroidism risk [36]. Notably, rare predicted loss-of-function variants in *NFATC1* confer a higher effect size for hypothyroidism than those observed in *TSHR* [36], highlighting the broader thyroid relevance of this locus. Additional support for this locus was provided by proteomic profiling. Six of the eleven proteins altered in affected family members (IL-7, CXCL1, CXCL5, CXCL6, TNFRSF9, and IL-12B) are involved in immune pathways associated with NFATC1, which is consistent with, although it does not establish, a functional role of this SV-enriched region (Supplemental Note S1).

The *PDCD6-AHRR* cluster, including a 40 bp insertion within a highly active enhancer alongside additional overlapping variants, may alter tissue-specific gene expression by disrupting chromatin architecture and distal enhancer activity. *PDCD6* contributes to apoptosis and cellular stress responses [37], which are processes relevant to immune regulation, while altered expression in this region has been linked to thyroid-related phenotypes [38]. Proteomic profiling was consistent with a possible biological relevance of this locus, with several altered proteins (SIRT2, ST1A1, STAMBP, and EN-RAGE) mapping to inflammatory, stress-response, and apoptotic pathways associated with the *PDCD6-AHRR* diminished activity (Supplemental Note S1).

The convergence of genomic and exploratory proteomic findings on the same biological modules makes these two regions priority candidates for functional follow-up. Finally, *AXIN1* provided a complementary example of proteomics-guided locus prioritization. Unlike the proteins linked to the *NFATC1-ATP9B* and *PDCD6-AHRR* loci, *AXIN1* displayed a pattern distinct from that observed in the LT4-HT patients. This observation prompted targeted re-analysis of the *AXIN1* genomic region, leading to the identification of two non-coding deletions overlapping putative regulatory elements supported by eQTL and chromatin interaction data. Although functional validation is required, the convergence of proteomic and genomic evidence supports *AXIN1* as a potential family-specific modifier locus.

A central observation supporting our integrated model is that all three sequenced affected family members exhibited polygenic risk scores within the top 5% (≥95th percentile) of the HT distribution, approximately two standard deviations above both unrelated HT cases and controls (Figure 2). In contrast, the unaffected father showed a PRS close to the population mean, suggesting that high polygenic load may contribute to disease manifestation in this pedigree. A polygenic burden of this magnitude has previously been reported to be comparable to that conferred by rare monogenic mutations in other conditions [10], suggesting that PRS alone may meaningfully lower the threshold for HT manifestation in genetically loaded individuals.

None of the SVs identified in this family was classified as likely pathogenic or pathogenic, and about 67% of the prioritized SVs are relatively common in reference populations (CoLoRS frequencies 0.017–0.27). Pathogenic relevance therefore cannot be attributed to any individual SV. This is expected for a common polygenic disease such as HT, in which genetic risk is carried predominantly by common variants of modest effect, as shown by the >150 loci identified by GWAS [20]. Under a liability-threshold model, such variants are expected to act collectively. Accordingly, the main observation of this study is not the effect of any single SV, but the non-random localisation of the SVs shared by the affected relatives at established hypothyroidism GWAS loci, inherited together with a high polygenic background. Whether this combination is functionally distinct from carrying any single variant remains to be tested.

Our approach aligns with recent breakthroughs in other complex traits, such as amyotrophic lateral sclerosis and schizophrenia, where small structural variants (SSVs) undetected by short-read sequencing have been proposed as a source of missing heritability [39,40,41]. Our observation of SV enrichment within known hypothyroidism GWAS loci offers a framework for interpreting non-coding GWAS signals. In line with recent fine-mapping studies, our data are consistent with the hypothesis that some thyroid-associated GWAS SNPs may tag structural variants that affect long-range gene regulation, which could be tested by fine-mapping and functional studies [42,43].

Taken together, our findings support a model in which a high polygenic burden may establish a sensitized genetic background upon which regulatory SVs may act as modifiers of gene expression in thyroid and immune pathways. Within this framework, the apparent autosomal dominant segregation observed in this family may represent a pseudo-Mendelian pattern arising from the co-inheritance of multiple risk factors that collectively exceed a pathogenic threshold.

The primary strength of this study lies in the integrated application of WES, LR-WGS, and PRS in a single multigenerational pedigree, which enabled the identification of segregating structural variation in loci where standard exome sequencing alone had failed to provide conclusive results. The combination of these data with hypothyroidism GWAS signals allowed us to propose a multi-hit model that may explain the pseudo-Mendelian segregation observed in this family through the synergy of structural alterations and high background polygenic burden.

Several limitations should be acknowledged. First, the candidate SVs were not functionally validated, and their regulatory impact is inferred from eQTL, Hi-C chromatin interaction and chromatin accessibility data. Functional validation of the proposed model is challenging, because a cumulative effect of several regulatory variants on a high polygenic background cannot be tested by perturbing individual variants, and some candidates, such as TRHDE, act within the hypothalamic–pituitary–thyroid axis, which would require whole-organism models. In addition, many human enhancers and lncRNAs, including LALTOP, are poorly conserved in rodents. Second, only three of the six affected family members were available for LR-WGS and PRS analysis; thus, sharing of an SV does not by itself establish co-segregation with disease. Additionally, the prioritized SVs could not be validated in the remaining family members, and generalization of the PRS observation to the full affected lineage requires further sequencing. Third, proteomic analysis was not powered enough given the limited sample size (*n* = 7 affected family members); these analyses are exploratory and intended solely to illustrate potential functional correlates of the identified structural variants. A minor technical limitation involves the difference in pre-centrifugation times between biobank controls (1–3 h) and on-site family samples (within 8 h), though potential confounding effects were minimized by placing the family samples immediately on ice. Finally, environmental exposures were not assessed.

In this multigenerational pedigree with early-onset HT, our findings are consistent with a multi-hit genetic architecture rather than a single monogenic cause. The genome-wide enrichment of segregating SVs at hypothyroidism GWAS loci represents the principal molecular observation and suggests that non-coding structural variation may represent an underrecognized regulatory layer in established disease-susceptibility regions; i.e., our findings suggest that part of the missing heritability in HT may reside in non-coding SVs, which are often overlooked in standard SNP-based studies. This regulatory layer is superimposed on an extreme polygenic burden (≥95th percentile) shared by all sequenced affected members, which together may lower the physiological threshold for disease manifestation. The convergence of these factors provides a plausible explanation for the pseudo-Mendelian segregation and pediatric onset observed in this family. Beyond the family-specific findings, the integrative analytical framework linking population-scale GWAS data with family-specific structural variation illustrates a generalizable strategy for dissecting the genetic architecture of complex autoimmune disorders. The prioritized loci, reported in full in the Supplementary Tables, provide specific hypotheses for future functional studies, such as reporter assays and CRISPR-based enhancer perturbation in thyroid and immune cell models, and for replication in independent families. Furthermore, exploratory proteomics in affected family members was consistent with two SV cluster loci and identified an additional candidate, illustrating the added value of multi-omic follow-up in genomic studies.

## Figures and Tables

**Table 1:** Baseline demographic, clinical, and biochemical characteristics of family members.

	HT (Yes/No)	WES/PacBioWGS	Age	BMI	Sex	Euthyrox Therapy	Duration of Disease (Days)	T3	T4	TSH	TgAb	TPOAb	fT3	fT4
1	yes	/	48.4	31.22	1	yes	4894	1.3	114	9.01	50.5	184	3.3	21.8
2	no	/	25.2	20.20	2	no	0	2.1	121	0.945	207	35.7	3.9	14.6
3	no	WES	64.9	23.92	1	no	0	1.7	108	5.61	131	1.3	3.3	11.6
4	yes	WES	26.9	19.60	2	yes	8182	1.9	128	1.83	3500	515	3.9	19.1
5	no	/	63.2	28.09	1	no	0	1.9	114	2.65	5	24.3	4.2	9.7
6	yes	/	61.9	21.16	2	yes	87	1.8	123	5.11	189	453	3.9	12.3
7	yes	WES/PacBio	32.7	21.47	2	yes	10,373	1.9	135	2.57	275	1706	3.9	14.8
8	no	WES/PacBio	37.4	23.15	1	no	0	1.9	121	2.43	5.9	1.8	4.5	13.3
9	yes	WES/PacBio	13.6	19.03	2	yes	1972	2.2	138	2.51	257	175	4.3	13.5
10	yes	WES	34.1	24.80	2	yes	3433	2.1	108	3.22	87	121	4.4	11.8
11	yes	WES/PacBio	52.5	22.15	2	yes	15,121	1.9	135	2.13	3346	271	4.4	15
12	no	WES	77.8	31.56	1	no	0	2.1	151	1.34	10.8	1	3.9	13.2
13	yes	WES	71.7	30.11	2	yes	5625	2.1	121	2.12	10.7	1.8	4.4	13.7
14	yes	/	51.3	25.20	1	yes	1607	1.8	123	1.69	2480	1720	3.9	17.4
15	no	/	15.4	22.41	2	no	0	2.2	115	1.36	12.6	7.5	4.7	14.8

**Table 2:** Summary of prioritized candidate loci and integrative regulatory evidence in target tissues relevant to Hashimoto’s thyroiditis.

Gene	Chromosome	Start	End	Length	Type	Frequency CoLoRSdb	Regulatory Element (RE)	eQTL	Intact Hi-C Loops	Affected Tissue/System by RE
*TPO*	2	1,493,494	1,493,566	−72	DEL	0.267344	EH38E3319998-Distal enhancer	ENSG00000283766, ENSG00000228613, TPO	N/A	Thyroid
*TPO*	2	1,511,227	1,511,343	−116	DEL	N/A	EH38E3320015-Distal enhancer	N/A	ENSG00000236665	Thymus-derived CD4-positive, alpha-beta T cell
*TPH2*	12	7.2 × 10^7^	7.2 × 10^7^	−503	DEL	0.0795375	EH38E1624299-CA-H3K4me3	TRHDE, TRHDE-AS1	N/A	Spinal cord, coronary artery, tibial artery
*~TSHR (+316 kb)*	14	8.1 × 10^7^	8.1 × 10^7^	−38	DEL	0.251577	NO	N/A	N/A	N/A
*ATP9B*	18	7.9 × 10^7^	7.9 × 10^7^	1583	INS	0.0171689	EH38E4188896-CA	ENSG00000279474, ENSG00000279077	N/A	Thyroid, brain
*NFATC1*	18	7.9 × 10^7^	7.9 × 10^7^	49	INS	0.0451997	EH38E3278273-Promoter	ENSG00000267287	N/A	Thyroid, brain
*NFATC1*	18	8 × 10^7^	8 × 10^7^	75	INS	0.0332866	EH38E3278311-Distal enhancer	N/A	ENSG00000286186, CNDP2, ATP9B, ZADH2, NFATC1, CTDP1, ENSG00000178412, ENSG00000274828, SLC66A2, RBFA, TXNL4A, ENSG00000267127, ZNF407, ZNF407-AS1, ADNP2, ENSG00000286245, HSBP1L1, ENSG00000287879	Lymphoid cell populations (T cells, B cells and NK cells) *
*NFATC1*	18	8 × 10^7^	8 × 10^7^	−263	DEL	N/A	EH38E3278313-Distal enhancer	N/A	ENSG00000286186, CNDP2, ATP9B, ZADH2, NFATC1, CTDP1, ENSG00000178412, ENSG00000274828, SLC66A2, RBFA, TXNL4A, ENSG00000267127, ZNF407, ZNF407-AS1, ADNP2, ENSG00000286245, HSBP1L1, ENSG00000287879	Lymphoid cell populations (T cells, B cells and NK cells) *
*NFATC1*	18	8 × 10^7^	8 × 10^7^	−184	DEL	N/A	EH38E3278314-Distal enhancer & EH38E3278315-Distal enhancer	N/A	ENSG00000286186, CNDP2, ATP9B, ZADH2, NFATC1, CTDP1, ENSG00000178412, ENSG00000274828, SLC66A2, RBFA, TXNL4A, ENSG00000267127, ZNF407, ZNF407-AS1, ADNP2, ENSG00000286245, HSBP1L1, ENSG00000287879	Lymphoid cell populations (T cells, B cells and NK cells) *
PDCD6-AHRR **	5	318,511	318,511	40	INS	N/A	EH38E2351870-Distal enhancer	SLC9A3, SLC9A3-AS1, EXOC3, PDCD6, PDCD6-DT, SDHA, EXOC3-AS1, CCDC127, AHRR, ENSG00000250848, ENSG00000261434	CEP72, CEP72-DT, EXOC3, EXOC3-AS1, CEP72, CEP72-DT, BRD9, TRIP13, ENSG00000271781, ZDHHC11B, ENSG00000288930	Thyroid, brain, whole blood
*127 kb upstream from AXIN1*	16	159,987	160,572	585	DEL	0.110722	EH38E4133037-CA & EH38E4133038-CA	AXIN1, FAM234A & HBZ	N/A	Brain
*NPRL3 (163 kb upstream from AXIN1)*	16	123,899	123,986	87	DEL	0.049054	EH38E4133026-Proximal Enhancer & EH38E4133027-Proximal Enhancer	NPRL3, WASIR2, IL9RP3, SNRNP25, ENSG00000269482 & WFIKKN1, NPRL3, SNRNP25, FAM234A, RHBDF1, MPG	HBQ1	N/A

Note. * CD4-positive alpha-beta memory T cell, naive CD4-positive alpha-beta T cell, CD4-positive alpha-beta T cell, CD8-positive alpha-beta T cell, natural killer cell, T-helper 2 cell, naive thymus-derived CD4-positive alpha-beta T cell, B cell, CD8-positive alpha-beta T cell, T-helper 2 cell, OCI-LY8; ** ChIA-PET Interactions: EXOC3, PP7080, SLC9A3-AS1.

## Data Availability

The data supporting the findings of this study are available upon reasonable request from the corresponding author.

## Supplementary Materials

The following supporting information can be downloaded at: https://www.mdpi.com/article/10.3390/genes17101303/s1, Figure S1: Stepwise filtering and prioritization of variants. (A) Whole-exome sequencing of nine family members. (B) Long-read whole-genome sequencing of four family members. Structural variants (SVs) present in the affected proband, mother and grandmother and absent in the unaffected father (±100 bp) were defined as affected-lineage-shared SVs (*n* = 2000) and prioritized through four complementary routes, resulting in the 12 SVs listed in Table 1. INS, insertion; DEL, deletion; BND, breakend; DUP, duplication; INV, inversion; VUS, variant of uncertain significance; LP/P, likely pathogenic/pathogenic; Figure S2: GTEx eQTL evidence supporting candidate regulatory regions affected by structural variants. Violin plots show significant associations between variants located within or near enhancer regions overlapping candidate structural variants and expression of nearby genes in relevant tissues. Panels display associations for *TRHDE* (a), *ENSG00000228613* and *ENSG00000283766* (b), *ENSG00000267287* (c), *ENSG00000279077* (d), *PDCD6-DT* (e), and *AXIN1* (f–g). Expression values represent normalized gene expression levels from GTEx, stratified by genotype. Sample sizes for each genotype group are indicated in parentheses, and *p*-values correspond to GTEx eQTL analyses; Table S1: Demographic and clinical characteristics of affected family members and controls; Table S2a: Overlap analysis between hypothyroidism GWAS SNPs and SV-defined genomic windows; Table S2b: Unique SV regions overlapping with hypothyroidism SNPs; Table S2c: Unique hypothyroidism GWAS SNPs overlapping with SV windows; Table S3: Rare heterozygous variants identified via familial segregation filtering of WES data; Table S4: Disease-segregating structural variants (SVs) identified by LR-WGS; Table S5: Disease-segregating structural variants (SVs) classified by AnnotSV as variants of uncertain significance (VUS); Supplementary Materials [16,17,22,23,24,34,35,36,37,38,44,45,46,47,48,49,50], Supplementary Note S1 [19,35,51,52,53,54,55,56,57,58,59,60], Supplementary Note S2 [29].

## Abbreviations

The following abbreviations are used in this manuscript:

HT	Hashimoto’s thyroiditis
WES	whole-exome sequencing
HiFi	high-fidelity
LR-WGS	long-read whole-genome sequencing
SV	structural variant
GWAS	genome-wide association study
lncRNA	long non-coding RNA
PRS	polygenic risk score
SNP	single-nucleotide polymorphism
CRO-HT	Croatian Hashimoto’s Thyroiditis Biobank
TSH	thyroid-stimulating hormone
FT4	free thyroxine
FT3	free triiodothyronine
TgAb	thyroglobulin antibodies
TPOAb	thyroid peroxidase antibodies
gDNA QC	genomic DNA quality control
FASTQ	FASTQ sequence file format
GRCh37/hg19	Genome Reference Consortium Human Build 37/human genome build 19
GATK	Genome Analysis Toolkit
MAF	minor allele frequency
5-HTP	5-hydroxytryptophan
TPH	tryptophan hydroxylase
HPT	hypothalamic–pituitary–thyroid
ELISA	enzyme-linked immunosorbent assay
SPSS	Statistical Package for the Social Sciences
CCS	Circular Consensus Sequencing
QV	quality value
GRCh38	Genome Reference Consortium Human Build 38
BAM	Binary Alignment Map
Bp	base pair
ACMG	American College of Medical Genetics and Genomics
ENCODE	Encyclopedia of DNA Elements
ChIA-PET	Chromatin Interaction Analysis by Paired-End Tag sequencing
eQTL	expression quantitative trait locus
GTEx	Genotype-Tissue Expression
LD	linkage disequilibrium
Kb	kilobase
CEU	Utah residents with Northern and Western European ancestry
LT4	levothyroxine
SNV	single-nucleotide variant
OR	odds ratio
CI	confidence interval
cCRE	candidate cis-regulatory element
3D	three-dimensional
Hi-C	high-throughput chromosome conformation capture
AF	allele frequency
SD	standard deviation
TRH	thyrotropin-releasing hormone
SSV	small structural variant
AI	artificial intelligence
RE	regulatory element
DEL	deletion
CD4	cluster of differentiation 4
INS	insertion
NK	natural killer
CD8	cluster of differentiation 8
