# Supplementary material for: Non-Coding Structural Variants in Regulatory Regions and High Polygenic Burden May Jointly Contribute to Early-Onset Familial Hashimoto’s Thyroiditis
Source: Genes (Basel). 2026 Oct 20;17(10):1303. doi: 10.3390/genes17101303 (PMC13704020; doi:10.3390/genes17101303)
Supplement: Supplementary file 1 [file genes-17-01303-s001.zip › Figure S1.pdf]

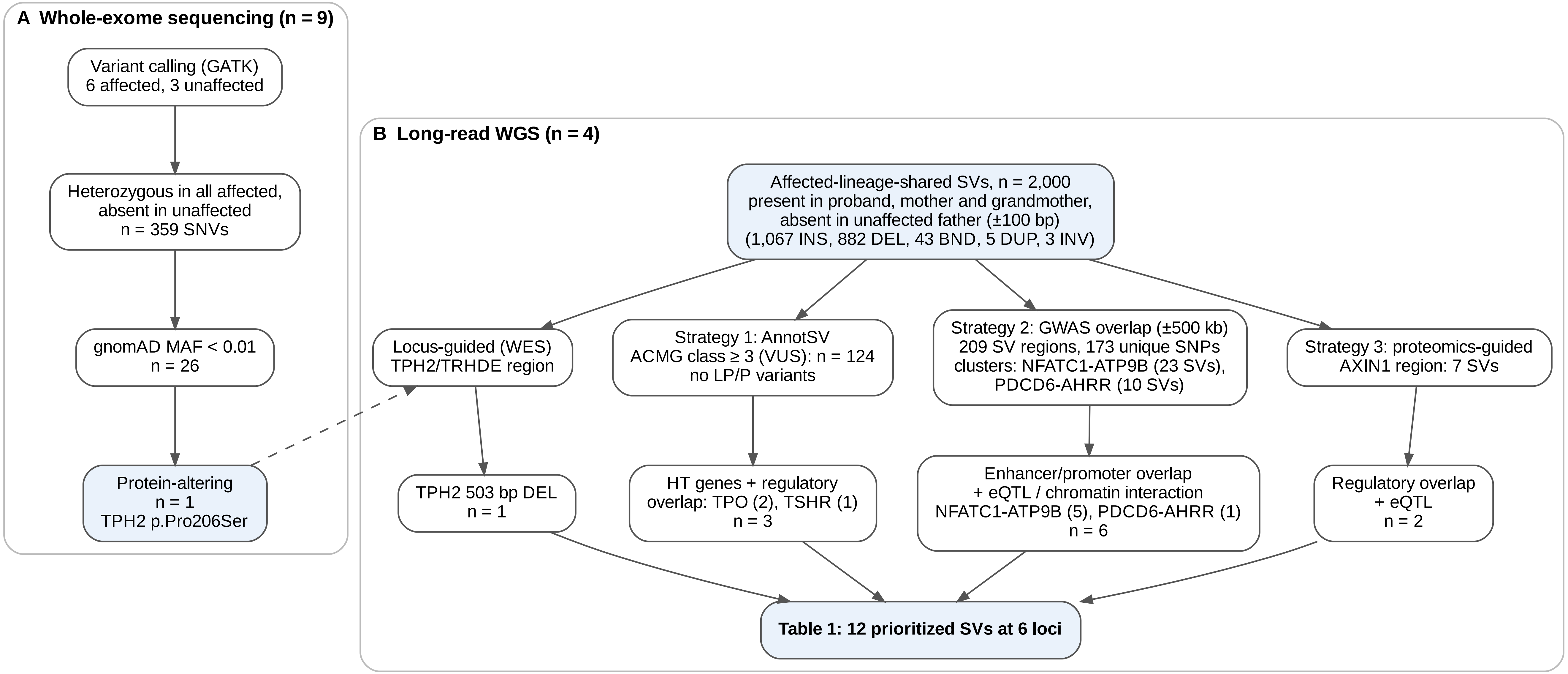
A Whole-exome sequencing (n = 9)
Variant calling (GATK)
6 affected, 3 unaffected
Heterozygous in all affected,
absent in unaffected
n = 359 SNVs
gnomAD MAF < 0.01
n = 26
Protein-altering
n = 1
TPH2 p.Pro206Ser
B Long-read WGS (n = 4)
Affected-lineage-shared SVs, n = 2,000
present in proband, mother and grandmother,
absent in unaffected father (±100 bp)
(1,067 INS, 882 DEL, 43 BND, 5 DUP, 3 INV)
Locus-guided (WES)
TPH2/TRHDE region
Strategy 1: AnnotSV
ACMG class ≥ 3 (VUS): n = 124
no LP/P variants
Strategy 2: GWAS overlap (±500 kb)
209 SV regions, 173 unique SNPs
clusters: NFATC1-ATP9B (23 SVs),
PDCD6-AHRR (10 SVs)
Strategy 3: proteomics-guided
AXIN1 region: 7 SVs
TPH2 503 bp DEL
n = 1
HT genes + regulatory
overlap: TPO (2), TSHR (1)
n = 3
Enhancer/promoter overlap
+ eQTL / chromatin interaction
NFATC1-ATP9B (5), PDCD6-AHRR (1)
n = 6
Regulatory overlap
+ eQTL
n = 2
Table 1: 12 prioritized SVs at 6 loci
