# Supplementary material for: Non-Coding Structural Variants in Regulatory Regions and High Polygenic Burden May Jointly Contribute to Early-Onset Familial Hashimoto’s Thyroiditis
Source: Genes (Basel). 2026 Oct 20;17(10):1303. doi: 10.3390/genes17101303 (PMC13704020; doi:10.3390/genes17101303)
Supplement: Supplementary file 1 [file genes-17-01303-s001.zip › Supplementary Materials.pdf]

A multi-hit genomic architecture driven by non-coding structural variants in regulatory regions underlies early-onset Hashimoto's thyroiditis

Vesna Boraska Perica[1], Ana Barić Žižić[2], Sanda Sladić[2], Marko Vuletić[2], Ivana Listeš[1], Dean Kaličanin[1], Vanna Žnidar[1], Vedrana Čikeš Čulić[3], Vesela Torlak Lovrić[2], Ante Punda[2], Veselin Škrabić[4], Maja Barbalić*[5]

[1] Department of Medical Biology, University of Split School of Medicine, Split, Croatia;
[2] Department of Nuclear Medicine, University Hospital of Split, Split, Croatia;
[3] Department of Chemistry and Biochemistry, University Hospital of Split, Split, Croatia;
[4] School of Medicine, University of Mostar, Mostar, BiH
[5] Department of Biology, University of Split Faculty of Science, Split, Croatia
* Corresponding author

Supplementary Materials

Supplementary Methods

WES annotation and filtering

Variant annotation included population frequency data from gnomAD v2.1.1 and basic variant information from public databases. Following initial variant calling, a total of 359 variants passed the segregation filter. After applying population frequency filtering (MAF < 0.01), 26 variants remained for further evaluation. The majority of these variants were located in non-coding regions without clear evidence of functional relevance. Only a single protein-altering coding variant, located in the *TPH2* gene, was identified and selected for further investigation.

Long-read whole genome sequencing (LR-WGS)

SMRTbell libraries were constructed from genomic DNA following standard PacBio protocols, including DNA damage repair, end-repair, A-tailing, and ligation of hairpin adapters. Libraries were purified using AMPure PB beads and prepared for sequencing by polymerase binding. Sequencing was performed using PacBio HiFi technology on the Sequel II/IIe platform (Novogene Co., Ltd.), generating highly accurate circular consensus sequencing (CCS) reads (QV ≥ 20). Reads were aligned to the human reference genome (GRCh38) using pbmm2.

Prioritisation of SVs

Prioritization of structural variants (SVs) was performed using the web version of the AnnotSV tool. The input file was constructed using selected SV genomic coordinates together with associated variant information obtained from the varvis® platform. Reference and alternative alleles were determined partly through overlaps with the CoLoRS database (https://colorsdb.org/). For variants where no overlap with the database was detected, the type of SV (e.g., deletion,

duplication) was used as the alternative allele. Additionally, four columns describing segregation within the family were added to the input table: Affected 1, Affected 2, and Affected 3 representing the affected family members, and Control representing the unaffected individual. The resulting table was converted into a VCF file using a server-based conversion approach and subsequently uploaded to AnnotSV for annotation and prioritization. AnnotSV annotates SVs by identifying overlaps with user-defined gene sets (RefSeq or ENSEMBL) and integrating diverse genomic features, including gene-specific clinical data (GenCC, OMIM, intolerance scores), population frequencies (DGV, 1000 Genomes), regulatory elements (promoters, enhancers, TADs), and relevant HPO terms (HP:0000872, HP:0025379, HP:0032069). Furthermore, the tool characterizes breakpoints via GC content and repeats, while providing systematic SV classification based on established ACMG and ClinGen guidelines (1,2). An overview of the WES and LR-WGS analytical workflows, including variant filtering and the three complementary SV prioritization strategies, is provided in Supplementary Figure S1.

Whole-genome genotype quality control and imputation

We used the Illumina Infinium Global Screening Array v3 for CROHT biobank participants (HT cases and controls). Sample-level QC included call rate filtering (<95%), sex concordance checks, heterozygosity outlier detection (±3 SD), and multidimensional scaling (MDS) to assess population stratification, resulting in the exclusion of four samples. Variant-level QC excluded SNPs with call rate <99%, minor allele frequency <5%, deviation from Hardy–Weinberg equilibrium (exact $p < 1\times10^{-4}$), or poor cluster separation (score ≤0.4), leaving 403 individuals (202 controls and 201 cases) and 487,147 SNPs for analysis. All analyses were performed on a Linux-based server using the bioinformatics tools PLINK v1.9 and PLINK v2.0, with data visualization conducted in R version 4.4.2. Following rigorous QC, an imputation of genotypes was performed via the Helmholtz Munich Imputation Server (https://imputationserver.helmholtz-munich.de/index.html#!), with the Haplotype Reference Consortium (HRC) reference panel, yielding a final dataset of approximately 39.1 million high-quality SNPs available for analysis.

Proxy selection for PRS calculation

To maximize the coverage of the HT-associated variants reported in the original GWAS (Bujnis et al., manuscript under review), we performed a proxy search for 21 variants that were not directly present in our imputed genotype data. Proxy variants were identified using the LDproxy tool from the LDlink suite, applying a linkage disequilibrium threshold of (R^2>0.8) based on the European population reference. While 13 suitable proxies met these criteria and were successfully included, the remaining eight variants were excluded for the following technical reasons: two variants had no proxies meeting the threshold, one was not available in the LDproxy database, and two had suitable proxies identified that were nonetheless absent from our genotype data. Furthermore, for three variants, LDproxy reported proxies for a different nearby variant at the same genomic position rather than for the target variant itself, leading to their exclusion to ensure data integrity.

Consequently, the final PRS was computed using a total of 147 high-quality variants, comprising 134 original GWAS variants and 13 successfully identified proxies.

Supplementary Results/Discussion

***TPH2*/*TRHDE* locus**

The identified 503 bp deletion overlaps the candidate cis-regulatory element EH38E1624299 located within an intron of the *TPH2* gene. While it exhibits predominantly low chromatin accessibility across most adult tissues, the presence of CTCF binding (Max Z-score = 1.81) suggests a significant structural role. ENCODE data indicate that this element likely functions as a topological anchor, facilitating long-range 3D chromatin looping to bridge the genomic distance between the *TPH2* locus and the neighboring region (3). To evaluate the functional consequences of this deletion, the lost genomic region was intersected with validated regulatory variants. Using the GTEx eQTL Calculator (4), rs1352251 was identified as a functional proxy within the deleted segment. The statistically significant association between the proxy eQTL variant and TRHDE expression in the spinal cord provides strong evidence that this element is indeed functional in the central nervous system.

Beyond the central nervous system, the EH38E1624299 enhancer exhibits highly dynamic, tissue-dependent regulatory activity. Our analysis of GTEx data identifies rs1352251 as a functional proxy within the deleted segment. In vascular tissues, the T allele (lost in the deletion) correlates with a reduction in TRHDE expression in the coronary artery ($P = 1.12 \times 10^{-2}$). In contrast, a robust but discordant eQTL effect was identified in the tibial artery ($p = 4.44\times 10^{-7}$), where the same allele significantly increases mRNA levels. The discrepancy between the relatively "quiet" epigenomic marks (low CA, low H3K27ac) and these highly significant eQTL p-values suggests that this element exerts its primary influence in specific cell types or under physiological triggers not fully captured by bulk tissue averages. Ultimately, the observed deletion likely triggers a 'dual-hit' transcriptional dysregulation in both neural and vascular environments, potentially contributing to the complex phenotype observed in the affected family members.

***TPO/LALTOP* locus**

The genomic region overlapping the 72 bp deletion (chr2:1,493,495–1,493,566) exhibits biochemical signatures consistent with regulatory activity across thyroid and multiple other tissues. According to the ENCODE Registry of cCREs and ReMap transcription factor datasets, the EH38E3319998 element demonstrates a DNase Max Z-score of 4.26, indicative of accessible chromatin architecture (ATAC score of 3.17), and holds an acetylation signature typical of active enhancers (H3K27ac Max Z-score = 2.94). Although none of the evaluated variants (rs77496745, rs67495882, rs114138459, rs28991270) lie within the deleted bases, two are directly adjacent (rs114138459 at +5 bp upstream; rs28991270 at +4 bp downstream), such that the deletion compresses their linear separation from ~78 bp to ~6 bp. This structural alteration potentially

alters local regulatory “grammar” within the enhancer-like sequence context (5,6). Systematic interrogation using the GTEx custom eQTL calculator for all tissues confirmed that regulatory perturbation at this locus is linked to transcriptional downregulation of ENSG00000283766 and ENSG00000228613 (LALTOP).

The second deletion (116-bp) spans 116 bp and overlaps both the *TPO* intron and the *LALTOP* transcript. This deletion additionally shortens lncRNA LALTOP by 0.1%. It partially overlaps enhancer EH38E3320015 (approximately 31%, 77 bp of this regulatory element is encompassed by the deleted interval), an active element marked by DNase hypersensitivity and H3K27ac histone marks. Hi-C data demonstrate that this enhancer forms a physical chromatin loop with the lncRNA *ENSG00000236665* in naive CD4+ T cells, suggesting its influence on T-cell function or differentiation and a role in maintaining immune homeostasis. *ENSG00000236665* is positioned inside the *SNTG2* gene that was recently associated with other thyroid conditions, a nodular goitre (7). Consequently, dysregulation of the EH38E3320015/ENSG0000023666*5* axis may disrupt T-cell tolerance mechanisms, which may be associated with the immune pathways underlying HT. The absence of this deletion in the CoLoRSdb database suggests that it represents a rare SV that may have a more pronounced effect.
According to ENCODE regulatory annotations, EH38E3320015 exhibits chromatin features consistent with enhancer activity, including DNase hypersensitivity and enrichment for H3K27ac histone marks.

***TSHR* locus**
A 38 bp deletion identified approximately 316 kb downstream of the *TSHR* gene was also considered during prioritization. Although this variant has a relatively high frequency in the CoLoRS database (0.252) and does not overlap any annotated regulatory element, it is noteworthy that a transcription factor binding region located between the variant and *TSHR* lies only 746 bp away from the variant and shows Intact Hi-C loop interactions with the *TSHR* locus. Nevertheless, the identified SV does not directly intersect a known regulatory element, and the available evidence is therefore insufficient to support a strong functional interpretation. At present, this variant can only be regarded as a weak candidate of uncertain significance, and any potential contribution to disease would require additional functional or genetic evidence.

***NFATC1/ATP9B* locus**
The significant enrichment of previously reported hypothyroidism GWAS signals within our SV regions suggests that these SVs are located in genomic 'hotspots' already linked to thyroid dysfunction. Interestingly, while the number of SNPs was enriched, the number of unique SV regions harboring these signals (n = 209) did not deviate from expectation, which indicates that the enrichment is driven by a high density of known risk variants within specific, highly relevant SV regions rather than a broad, low-density distribution across all detected SVs.

The 23 SVs identified in the *NFATC1/ATP9B* locus, although all located within intronic regions, may have a cumulative effect on its expression. Specifically, several SVs overlap with different regulatory elements and may potentially modulate their activity.

A 49-bp insertion warrants particular attention due to its location within the distal enhancer EH38E3278273, which functions as a promoter in B cells but also acts as an eQTL for a thyroid-expressed lncRNA (ENSG00000267287). Variants in close proximity to this insertion, including rs1660155 (positioned 19 bp upstream), exhibit statistically significant eQTL associations with the lncRNA ENSG00000267287 in thyroid tissue, supporting a potential regulatory effect.

Three additional SVs (deletions of 263 bp and 184 bp and one 75 bp insertion) overlap distal enhancers (EH38E3278311, EH38E3278313, EH38E3278314 and EH38E3278315) that exhibit chromatin interaction data, demonstrating Hi-C looping not only with the *NFATC1* gene itself but also with regulatory regions active in immune cell populations, including B, T, and NK cells. This suggests that structural perturbations within this region could have broader effects on immune-related regulatory networks.

NFATC1 encodes a calcium–calcineurin–dependent transcription factor that plays a central role in inducible gene expression during immune responses, particularly in T lymphocytes, where it regulates activation and cytokine production following T cell receptor signaling (8). In addition, NFATC1 activity has been shown to be critical for B cell development, further supporting its essential role in adaptive immunity (9).

NFATC1 has already been linked to autoimmunity, with transcriptomic differences reported between individuals with HT and controls (10). Its role is further emphasized by GWAS and PRS analyses, where rare predicted loss-of-function variants in *NFATC1* were significantly associated with an increased risk of hypothyroidism, showing a higher effect size than those observed for *TSHR*, suggesting a potentially strong effect in disease development (11).

The co-inheritance of this SV cluster across three generations aligns with the strong genetic signal for *NFATC1* reported in recent meta-analyses (11). While a cumulative effect of the entire cluster remains possible, this enhancer-associated insertion stands out as a plausible candidate for the observed association.

***PDCD6-AHRR* locus**

While the original GWAS-associated SNP is located within the *TPP* gene, multiple SVs were identified in the adjacent region, *PDCD6–AHRR*. These variants overlap multiple distal enhancer elements supported by eQTL signals and chromatin interaction data (Hi-C and ChIA-PET), suggesting their influence on chromatin architecture and long-range gene regulation.

A 40 bp insertion overlapping enhancer EH38E2351870 emerges as a strong candidate, as this enhancer shows extensive regulatory activity, with eQTL associations involving a relatively large number of genes across multiple tissues. The observation that several of these associations are significant in biologically relevant tissues, including the thyroid and whole blood (immune cells), supports the hypothesis that this variant may contribute to disease risk through modulation of gene expression in a tissue-specific manner. Given the presence of multiple overlapping SVs within the same locus, a combinatorial or additive regulatory effect is also possible.

PDCD6 encodes programmed cell death protein 6, which is involved in the regulation of numerous cellular processes, most notably apoptosis and alternative splicing (12). Although it has been primarily associated with tumorigenesis (13), it has been found to be significantly increased in the hippocampus of rats with congenital hypothyroidism (14).
Collectively, these findings raise the possibility that structural variation within the *PDCD6–AHRR* locus may contribute to disease by altering the coordinated regulation of genes involved in apoptosis and immune-related signaling

PTPRN2 locus
Among the 2000 affected-segregating SVs, 24 mapped to the *PTPRN2* locus, a gene previously associated with thyroid peroxidase antibody (TPOAb) levels (15). Several of these SVs were absent from the CoLoRS database, suggesting that they may represent rare or insufficiently characterized structural variants. Seven SVs overlapped regulatory elements, including candidate cis-regulatory elements (cCREs) and enhancer regions.
Two of these regulatory intervals contained significant eQTLs associated with expression of the *NCAPG2* gene in brain tissues, a gene that has been implicated in tumour progression across multiple cancer types and has recently been associated with immune cell infiltration and regulation of the tumour immune microenvironment (16). Specifically, rs764363561, located only 2 bp upstream of one of the identified SVs, was associated with *NCAPG2* expression in the cerebellar hemisphere ($P = 3.43 \times 10^{-3}$), while rs1638742, located 124 bp upstream of another SV, was associated with *NCAPG2* expression in the caudate nucleus (basal ganglia) ($P = 1.39 \times 10^{-2}$). The close proximity of these eQTLs to the identified SVs supports the functional relevance of the affected regulatory regions. However, our primary focus remains on the *PTPRN2* gene, given its enrolment in the HPT axis. Beyond its association with TPOAb levels, *PTPRN2* is of particular interest due to its role in neuroendocrine secretory processes. *PTPRN2* encodes a membrane-associated protein of dense-core secretory granules that is expressed in neuroendocrine tissues and plays an important role in secretory granule biogenesis and hormone secretion (17). Interestingly, another locus identified in this study is *TRHDE*, a gene encoding the thyrotropin-releasing hormone-degrading enzyme. While no direct interaction between *PTPRN2* and *TRHDE* has been reported, both genes participate in neuroendocrine pathways that could influence hypothalamic–pituitary signalling through distinct mechanisms. Therefore, the accumulation of SVs within regulatory regions of *PTPRN2* may represent an additional layer of genetic variation potentially relevant to neuroendocrine regulation and thyroid disease susceptibility, although functional validation will be required to determine their biological significance.

HLA locus
Finally, two breakends (BND) calls 15 bp apart in the HLA class I region (chr6:29,846,901 and chr6:29,846,916) likely represent fragments of a single complex rearrangement that short-read sequencing systematically misses owing to extreme HLA polymorphism. The HLA region is the strongest known autoimmune-disease susceptibility locus genome-wide, and any evidence of HLA

structural variation in a long-read dataset is noteworthy. IGV-level verification of the underlying read alignments is required before this signal can be considered a true positive.

Reference List:

1. Riggs ER, Andersen EF, Cherry AM, Kantarci S, Kearney H, Patel A, et al. Technical standards for the interpretation and reporting of constitutional copy-number variants: a joint consensus recommendation of the American College of Medical Genetics and Genomics (ACMG) and the Clinical Genome Resource (ClinGen). Genet Med. 2020 Feb;22(2):245–57. doi:10.1038/s41436-019-0686-8 PubMed PMID: 31690835.
2. Richards S, Aziz N, Bale S, Bick D, Das S, Gastier-Foster J, et al. Standards and guidelines for the interpretation of sequence variants: a joint consensus recommendation of the American College of Medical Genetics and Genomics and the Association for Molecular Pathology. Genet Med. 2015 May;17(5):405–24. doi:10.1038/gim.2015.30 PubMed PMID: 25741868.
3. ENCODE Project Consortium, Moore JE, Purcaro MJ, Pratt HE, Epstein CB, Shoresh N, et al. Expanded encyclopaedias of DNA elements in the human and mouse genomes. Nature. 2020 Jul;583(7818):699–710. doi:10.1038/s41586-020-2493-4 PubMed PMID: 32728249.
4. GTEx Consortium. The GTEx Consortium atlas of genetic regulatory effects across human tissues. Science. 2020 Sep 11;369(6509):1318–30. doi:10.1126/science.aaz1776 PubMed PMID: 32913098.
5. Jindal GA, Farley EK. Enhancer grammar in development, evolution, and disease: dependencies and interplay. Dev Cell. 2021 Mar 8;56(5):575–87. doi:10.1016/j.devcel.2021.02.016 PubMed PMID: 33689769.
6. Plank JL, Dean A. Enhancer function: mechanistic and genome-wide insights come together. Mol Cell. 2014 Jul 3;55(1):5–14. doi:10.1016/j.molcel.2014.06.015 PubMed PMID: 24996062.
7. White SL, Brasher MS, Pattee J, Zhou W, Chapman S, Jee YH, et al. Global multi-ancestry genome-wide analyses identify genes and biological pathways associated with thyroid cancer and benign thyroid diseases. Nat Genet. 2026 Feb;58(2):307–16. doi:10.1038/s41588-025-02483-w PubMed PMID: 41644669.
8. Hogan PG. Calcium-NFAT transcriptional signalling in T cell activation and T cell exhaustion. Cell Calcium. 2017 May;63:66–9. doi:10.1016/j.ceca.2017.01.014 PubMed PMID: 28153342.
9. Giampaolo S, Wójcik G, Klein-Hessling S, Serfling E, Patra AK. B cell development is critically dependent on NFATc1 activity. Cell Mol Immunol. 2019 May;16(5):508–20. doi:10.1038/s41423-018-0052-9 PubMed PMID: 29907883.
10. Xu X, Ma Y, Fan W, Wang W, Xu Z, Yang J. Comparative Transcriptomic Analysis of Pathogenic Mechanisms in Papillary Thyroid Carcinoma and Hashimoto's Thyroiditis. 2025. doi:10.21203/rs.3.rs-6689784/v1
11. Rand SA, Ahlberg G, Tragante V, Monfort LM, Zheng C, Feldt-Rasmussen U, et al. Genome-wide association study and polygenic risk prediction of hypothyroidism. Nat

Genet. 2025 Dec;57(12):3007–15. doi:10.1038/s41588-025-02410-z PubMed PMID: 41238958.
12. Zhu Y, Li Q. Multifaceted roles of PDCD6 both within and outside the cell. J Cell Physiol. 2024 May;239(5):e31235. doi:10.1002/jcp.31235 PubMed PMID: 38436472.
13. Wen SY, Liu YT, Wei BY, Ma JQ, Chen YY. PDCD6 Promotes Hepatocellular Carcinoma Cell Proliferation and Metastasis through the AKT/GSK3β/β-catenin Pathway. Biomed Environ Sci. 2023 Mar 20;36(3):241–52. doi:10.3967/bes2023.027 PubMed PMID: 37005078.
14. Li W, Song D, Sun Y, Lv Y, Lv J. microRNA-124-3p inhibits the progression of congenital hypothyroidism via targeting programmed cell death protein 6. Exp Ther Med. 2018 Jun;15(6):5001–6. doi:10.3892/etm.2018.6062 PubMed PMID: 29805523.
15. Matana A, Popović M, Boutin T, Torlak V, Brdar D, Gunjača I, et al. Genome-wide meta-analysis identifies novel gender specific loci associated with thyroid antibodies level in Croatians. Genomics. 2019 Jul;111(4):737–43. doi:10.1016/j.ygeno.2018.04.012 PubMed PMID: 29678681.
16. Wang Q, Li Z, Zhou S, Li Z, Huang X, He Y, et al. NCAPG2 could be an immunological and prognostic biomarker: From pan-cancer analysis to pancreatic cancer validation. Front Immunol. 2023;14:1097403. doi:10.3389/fimmu.2023.1097403 PubMed PMID: 36776838.
17. Stojilkovic SS, Sokanovic SJ, Constantin S. What is known and unknown about the role of neuroendocrine genes Ptprn and Ptprn2. Front Endocrinol (Lausanne). 2025;16:1531723. doi:10.3389/fendo.2025.1531723 PubMed PMID: 39926347.
