# Supplementary material for: Non-Coding Structural Variants in Regulatory Regions and High Polygenic Burden May Jointly Contribute to Early-Onset Familial Hashimoto’s Thyroiditis
Source: Genes (Basel). 2026 Oct 20;17(10):1303. doi: 10.3390/genes17101303 (PMC13704020; doi:10.3390/genes17101303)
Supplement: Supplementary file 1 [file genes-17-01303-s001.zip › Supplementary Note S1.pdf]

Supplementary Note S1

Exploratory plasma proteomic profiling in affected family members: *preliminary functional correlates of the identified genomic architecture*

Scope and interpretation. The analyses presented in this Supplementary Note 1 are exploratory and hypothesis-generating, derived from n = 7 affected family members. They are presented to illustrate potential functional correlates of the structural variants identified in the main manuscript. None of the findings presented here are independently powered discovery claims.

SN1. Methods

SN1.1 Study participants

Affected family members (n = 7) and population-based controls (n = 209) from the CRO-HT biobank (1) were included in proteomic profiling. For the comparative analysis, a group of unrelated HT patients on stable levothyroxine (LT4) replacement therapy confirmed euthyroid at sampling (EUTHY-LT4; n = 150]; TSH within reference range) was profiled using the identical Olink panel under the same pre-analytical conditions (1).

SN1.2 Olink proximity extension assay

Plasma protein levels were quantified using the Olink Target 96 Inflammation panel (Olink Proteomics AB, Uppsala, Sweden), measuring 92 inflammation-associated proteins by proximity extension assay (PEA) technology (2). Normalized Protein eXpression (NPX) values were used throughout. All assays were performed at the Olink service laboratory.

SN1.3 Statistical model

Differential protein analysis between affected family members and controls, and between EUTHY-LT4 patients and controls, was performed using linear regression with the following covariates: age, sex, and BMI. Multiple testing correction used the Benjamini–Hochberg false discovery rate (FDR) procedure; FDR $p < 0.05$ was the significance threshold. Analyses were performed in R using the *OlinkAnalyze* package.

SN2. Results

SN2.1 Significant plasma proteins that overlap in affected family members and HT patients of LT4 therapy

Eleven proteins reached FDR-adjusted significance ($p < 0.05$) in affected family members versus population-based controls (Supplementary Table SN1). Nine were reduced, and two were elevated. The largest reduction was observed for SIRT2 (mean NPX difference: −2.76; FDR $p = 1.78 \times 10^{-4}$). Of the 11 proteins, ten group into two clusters that map directly to the two main genomic

regions identified in the SV cluster analysis: six proteins link to the *NFATC1-ATP9B* locus (CXCL1, CXCL5, CXCL6, IL-7, TNFRSF9, IL-12B) and four to the *PDCD6–AHRR* locus (SIRT2, ST1A1, STAMBP, EN-RAGE). This correspondence is unlikely to arise by chance: of 92 proteins on the Olink panel, ten converge on two genomic regions independently identified through SV analysis and GWAS enrichment, suggesting that the SV clusters at these loci exert measurable, coordinated effects on downstream protein expression.

AXIN1 is the eleventh protein and is discussed separately in Section SN2.2. Full data and mechanistic assignments are provided in Supplementary Table SN1;

SN2.2 One family-specific signal

Comparison of affected family members with the EUTHY-LT4 group identified one protein with a pattern that is qualitatively distinct from the generic HT pathophysiology of patients treated with LT4 (Supplementary Table SN2). This comparison is critical because it allows discrimination of family-specific signals from effects attributable to disease burden or thyroid hormone replacement.

AXIN1 showed a 9.6-fold larger reduction in the affected family (−2.51 NPX; adjusted $P = 6.52 \times 10^{-4}$) versus EUTHY-LT4 patients (−0.26 NPX; $p = 0.39$, non-significant), confirming this is not a disease or treatment effect. Proteomics-guided targeted genomic follow-up identified two candidate SVs: a 585 bp deletion at chr16:159,987–160,572 (~127 kb upstream of *AXIN1*; absent from CoLoRS) overlapping two ENCODE Candidate Activator cCREs (EH38E4133037, EH38E4133038) with ChIA-PET chromatin interactions and a GTEx eQTL for *AXIN1* expression in whole blood (rs868300042). A second SV (87 bp deletion, chr16:123,899–123,986; CoLoRS AF = 0.049) overlaps two proximal enhancers (EH38E4133026, EH38E4133027) with ChIA-PET interactions but without a direct *AXIN1* eQTL signal. The remaining five SVs lack regulatory annotation. This locus is reported as a prioritized candidate in the main manuscript (Prioritization strategy 3).

Supplementary Table SN1. *FDR-significant proteins from the Olink Target 96 Inflammation panel. NPX = Normalized Protein eXpression. Difference = mean (affected family, n=7) − mean (controls, n=209). All models adjusted for age, sex, BMI, TSH, statin, smoking, diabetes, RE, OPA. Genomic locus assignments reflect the primary SV cluster with the strongest regulatory evidence;. ‡ EN-RAGE is elevated (not reduced); its directional reversal reflects loss of AHRR-mediated suppression of the S100A12 gene cluster.*

Protein	Mean (Family)	Mean (Controls)	Diff (F−C)	Adjusted P	Genomic locus	Proposed mechanistic link
SIRT2	3.84	6.60	−2.76	1.8×10^{-4}	PDCD6–AHRR	Deacetylates NF-κB p65 K310; reduced SIRT2 → sustained NF-κB activity

CXCL1	8.20	10.38	−2.17	3.8×10^{-6}	NFATC1/ATP9B	NFATc1 transcriptional target; neutrophil and immune cell chemoattractant
CXCL5	9.88	12.13	−2.25	3.1×10^{-5}	NFATC1/ATP9B	NFATc1 target; ELR^{+} chemokine; neutrophil trafficking and T/B cell homing
CXCL6	6.72	8.55	−1.83	2.9×10^{-4}	NFATC1/ATP9B	NFATc1 target; T/B cell-mediated immunity and lymphoid chemotaxis
IL-7	0.80	1.93	−1.13	3.0×10^{-4}	NFATC1/ATP9B	NFATc1-dependent pro-B to pre-B transition; NFATc1 deficiency blocks this step
AXIN1	2.78	5.28	−2.51	6.5×10^{-4}	AXIN1	Wnt/β-catenin scaffold; 9.6× larger reduction than in LT4-treated HT; cis-regulatory SV confirmed (see SN2.3)
ST1A1	6.91	8.64	−1.74	6.5×10^{-4}	PDCD6–AHRR	AHR/XRE target; AHRR SV disruption shifts AHR–AHRR balance → reduced ST1A1 transcription
STAMBP	4.83	7.12	−2.29	6.5×10^{-4}	PDCD6–AHRR	Deubiquitinase co-regulated with PDCD6 in apoptotic signalling
TNFRSF9	6.32	5.69	+0.63	3.1×10^{-3}	NFATC1/ATP9B	CD137; T cell co-stimulatory receptor; NFATc1 regulates T cell effector programme
EN-RAGE ‡	3.17	2.07	+1.09	2.6×10^{-2}	PDCD6–AHRR	AHRR actively suppresses S100A12; SV disruption releases suppression → elevated EN-RAGE (opposite of SIRT2/ST1A1/STAMBP; see SN3.2)
IL-12B	7.10	6.36	+0.74	3.1×10^{-2}	NFATC1/ATP9B	IL-12p40; Th1-polarising cytokine;

						NFATc1 cytokine regulation

Abbreviations: SIRT2, sirtuin 2; CXCL, C-X-C motif chemokine ligand; IL, interleukin; AXIN1, axis inhibition protein 1; ST1A1/SULT1A1, sulfotransferase 1A1; STAMBP, STAM-binding protein; TNFRSF9/CD137, TNF receptor superfamily member 9; EN-RAGE/S100A12, extracellular newly identified RAGE-binding protein; NPX, Normalized Protein eXpression.

SN3. Biological rationale for genomic–proteomic associations

SN3.1 NFATC1-ATP9B locus: CXCL1, CXCL5, CXCL6, IL-7, TNFRSF9, IL-12B

The *NFATC1-ATP9B* locus harbours the largest SV cluster in the primary analysis (23 SVs overlapping three GWAS hits and multiple enhancer elements), and six of the 11 FDR-significant proteins map to this locus, providing the strongest proteomics-to-genomics correspondence in the study. NFATc1 is a master transcription factor for T and B lymphocyte activation (3), CXCL1, CXCL5, and CXCL6 are canonical NFATc1 transcriptional targets of the ELR$^+$ CXC chemokine family, regulating neutrophil recruitment and lymphocyte trafficking in autoimmune settings (4). IL-7 drives the NFATc1-dependent pro-B to pre-B cell transition, a step blocked by NFATc1 deficiency (5). NFATc1 directly controls cytokine and chemokine gene expression in CD8+ T cells (6). All four were markedly reduced, consistent with impaired NFATc1-driven innate chemokine output. TNFRSF9 (CD137/4-1BB), a T cell co-stimulatory receptor whose effector gene program is regulated by NFATc1 (6), was elevated, as was IL-12B, a Th1-polarising cytokine modulated by NFATc1 (3).

Taken together, for the *NFATC1/ATP9B* locus, the coordinated reduction of three canonical NFATc1 target chemokines (CXCL1, CXCL5, CXCL6) together with reduced IL-7 is consistent with impaired NFATc1-driven immune gene expression, while the simultaneous elevation of TNFRSF9 and IL-12B suggests a compensatory shift toward T cell co-stimulatory and Th1-polarising programs. Our proteomic data suggest that structural disruption of NFATC1 cis-regulatory architecture may phenocopy the molecular consequences of coding variants at this locus, providing a mechanistic explanation for how non-coding SVs at this GWAS locus could contribute to disease susceptibility in the general population.

SN3.2 *PDCD6–AHRR* locus: SIRT2, ST1A1, STAMBP (reduced) and EN-RAGE (elevated)

The *PDCD6–AHRR* locus harbours 10 SVs overlapping four distal enhancer elements. Four proteins map to this locus, but through two mechanistically distinct pathways that must be considered separately.

Reduced proteins: SIRT2, ST1A1, STAMBP: These three proteins are reduced in affected family members in a direction consistent with diminished regulatory output at this locus. SIRT2 is a NAD+-dependent deacetylase that restrains NF-κB inflammatory transcription by deacetylating

p65 at K310 (7); its marked reduction (−2.76 NPX) is consistent with sustained NF-κB activity. ST1A1 is an AHR/XRE pathway target whose reduction is compatible with AHRR enhancer disruption, shifting the AHR–AHRR balance toward unrestricted AHR activity (8). STAMBP, a deubiquitinase in endosomal sorting and cellular stress, is functionally co-regulated with PDCD6 in apoptotic signalling. All three are also reduced in the general HT population (1), indicating that part of their signal reflects HT pathophysiology broadly, amplified in the family context by the accumulated SVs at this locus.

Elevated protein: EN-RAGE (S100A12) is mechanistically distinct: EN-RAGE is elevated in affected family members (+1.09 NPX; FDR p = 0.026) while being significantly reduced in the general HT population (−0.38 NPX; FDR p = 0.0008 in (1)). This directional reversal is not an inconsistency but reflects a fundamentally different type of regulatory relationship with the *PDCD6–AHRR* locus. AHRR does not activate S100A12, whereas it actively suppresses it. A recent study demonstrated that AHRR specifically restrains S100 protein expression and innate immune antimicrobial genes in immune cells independently of its canonical AHR-suppressive role (9). When AHRR enhancers are disrupted by SVs, this suppression is lost, and S100A12 expression is released, thus producing an elevation rather than a reduction. The absence of any disease-segregating SV at the chr1q21 S100A12 locus (Section SN2.3) confirms that the elevation is trans-regulatory rather than driven by local genetic variation. Accordingly, EN-RAGE and SIRT2/ST1A1/STAMBP represent two complementary downstream consequences of the same upstream AHRR disruption: reduction of AHRR-activated effectors and release of an AHRR-suppressed gene. EN-RAGE is a granulocyte-specific DAMP that amplifies innate immune responses through RAGE and TLR4-mediated NF-κB and MAPK activation (10,11), and its elevation would be expected to further sustain the pro-inflammatory state already indicated by reduced SIRT2.

Taken together, for the *PDCD6–AHRR* locus, the EN-RAGE (S100A12) directional reversal, elevated in the family and reduced in the general HT population, is particularly informative because it demonstrates a functional consequence of AHRR enhancer disruption not predicted by HT disease status alone. AHRR restrains the S100 gene cluster through active transcriptional suppression independently of the thyroid hormone axis; its disruption therefore produces a protein expression change mechanistically grounded in the specific regulatory architecture of this locus. The three simultaneously reduced proteins (SIRT2, ST1A1, STAMBP) and the one elevated protein (EN-RAGE) together potentially illustrate how a single SV cluster can produce bidirectional downstream effects — reducing AHRR-activated genes while releasing AHRR-suppressed ones — providing a richer functional portrait of locus disruption than any single protein direction could convey.

SN3.3 *AXIN1* locus

The only family-specific protein, AX1N1, and its corresponding *AXIN1* locus illustrate a complementary logic: the same biological perturbation, reduced Wnt/β-catenin scaffold activity,

is nominally present in the general HT population but below the significance threshold, and becomes statistically manifest only in the family where a rare regulatory deletion is segregating. Two independent deletions segregating in the affected family disrupt multiple regulatory elements within the *AXIN1* locus. The presence of significant AXIN1-associated eQTLs (rs868300042 and rs17146023) within the enhancers supports the regulatory relevance of these regions and suggests that the identified structural variants may influence AXIN1 expression through alteration of enhancer activity. This pattern is consistent with the broader concept that GWAS signals may represent population-level averages partly driven by rare, large-effect variants diluted across large samples (39,40). *AXIN1* encodes a central scaffold of the Wnt/β-catenin pathway with additional roles in Hippo, TGFβ, AMPK, and MAPK signalling, and its dysregulation has been implicated in thyroid neoplasia (12) and immune cell activation.

SN4. References

1. Žnidar V, Kaličanin D, Boban Z, Barić Žižić A, Vuletić M, Sladić S, et al. Inflammatory protein expression patterns in Hashimoto's thyroiditis: a cross-sectional observational study. Clin Proteomics. 2026 Mar 2;23(1). doi:10.1186/s12014-026-09593-5 PubMed PMID: 41772456.
2. Assarsson E, Lundberg M, Holmquist G, Björkesten J, Thorsen SB, Ekman D, et al. Homogenous 96-plex PEA immunoassay exhibiting high sensitivity, specificity, and excellent scalability. PLoS One. 2014;9(4):e95192. doi:10.1371/journal.pone.0095192 PubMed PMID: 24755770.
3. Müller MR, Rao A. NFAT, immunity and cancer: a transcription factor comes of age. Nat Rev Immunol. 2010 Sep;10(9):645–56. doi:10.1038/nri2818 PubMed PMID: 20725108.
4. Kimura H, Caturegli P. Chemokine orchestration of autoimmune thyroiditis. Thyroid. 2007 Oct;17(10):1005–11. doi:10.1089/thy.2007.0267 PubMed PMID: 17910527.
5. Giampaolo S, Wójcik G, Klein-Hessling S, Serfling E, Patra AK. B cell development is critically dependent on NFATc1 activity. Cell Mol Immunol. 2019 May 15;16(5):508–20. doi:10.1038/s41423-018-0052-9
6. Klein-Hessling S, Muhammad K, Klein M, Pusch T, Rudolf R, Flöter J, et al. NFATc1 controls the cytotoxicity of CD8+ T cells. Nat Commun. 2017 Sep 11;8(1):511. doi:10.1038/s41467-017-00612-6
7. Rothgiesser KM, Erener S, Waibel S, Lüscher B, Hottiger MO. SIRT2 regulates NF-κB-dependent gene expression through deacetylation of p65 Lys310. J Cell Sci. 2010 Dec 15;123(24):4251–8. doi:10.1242/jcs.073783
8. Bock KW. Aryl hydrocarbon receptor (AHR): From selected human target genes and crosstalk with transcription factors to multiple AHR functions. Biochem Pharmacol. 2019 Oct;168:65–70. doi:10.1016/j.bcp.2019.06.015 PubMed PMID: 31228464.
9. Weighardt H, Shapiro M, Mayer M, Förster I, Stockinger B, Diny NL. The AHR repressor limits expression of antimicrobial genes but not AHR-dependent genes in intestinal eosinophils. J Leukoc Biol. 2024 Jul 25;116(2):369–78. doi:10.1093/jleuko/qiae105 PubMed PMID: 38701199.
10. Viemann D, Strey A, Janning A, Jurk K, Klimmek K, Vogl T, et al. Myeloid-related proteins 8 and 14 induce a specific inflammatory response in human microvascular endothelial cells. Blood. 2005 Apr 1;105(7):2955–62. doi:10.1182/blood-2004-07-2520 PubMed PMID: 15598812.
11. Foell D, Frosch M, Sorg C, Roth J. Phagocyte-specific calcium-binding S100 proteins as clinical laboratory markers of inflammation. Clin Chim Acta. 2004 Jun;344(1–2):37–51. doi:10.1016/j.cccn.2004.02.023 PubMed PMID: 15149869.
12. Garcia-Rostan G, Camp RL, Herrero A, Carcangiu ML, Rimm DL, Tallini G. Beta-catenin dysregulation in thyroid neoplasms: down-regulation, aberrant nuclear expression, and CTNNB1 exon 3 mutations are markers for aggressive tumor phenotypes and poor prognosis. Am J Pathol. 2001 Mar;158(3):987–96. doi:10.1016/s0002-9440(10)64045-x PubMed PMID: 11238046.
