# Supplementary material for: Non-Coding Structural Variants in Regulatory Regions and High Polygenic Burden May Jointly Contribute to Early-Onset Familial Hashimoto’s Thyroiditis
Source: Genes (Basel). 2026 Oct 20;17(10):1303. doi: 10.3390/genes17101303 (PMC13704020; doi:10.3390/genes17101303)
Supplement: Supplementary file 1 [file genes-17-01303-s001.zip › Supplementary Note S2.pdf]

Supplementary Note S2

Exploratory NMR metabolomic profiling in affected family members

Materials & Methods

NMR metabolomic profiling

Plasma metabolomic profiling was performed using a targeted NMR-based platform (Nightingale Health, Helsinki, Finland), quantifying 250 metabolic measures including lipoprotein subclass concentrations and compositions, fatty acids, amino acids, and glycolytic metabolites. Profiling was conducted in all sequenced affected family members (n=7) and in a subset of the CRO-HT biobank comprising untreated hypothyroid (HYPO, n=140), euthyroid (EUTHY, n=192), and levothyroxine-treated (LT4, n=181) HT patients, as well as healthy controls. To isolate a family-specific metabolic signal, affected Murter family members were compared directly against the LT4 group, with adjustment for age, sex, BMI, statin use, levothyroxine dose per kg body weight, smoking status, diabetes, oral contraceptive use, and menopausal status using linear regression. Given the small sample size of the Murter cohort (n=7), all findings from this comparison are interpreted as directional hypotheses rather than definitive associations.

Results

Metabolomic profiling

To complement the genomic findings, plasma NMR metabolomic profiling was performed in affected family members and a population-based HT cohort. In treatment-naive comparisons, XXL_VLDL cholesterol fractions were robustly elevated in HT patients relative to controls independently of levothyroxine therapy, consistent with a disease-intrinsic compositional shift in the largest VLDL particles.

Direct comparison of Murter family members against unrelated LT4-treated HT patients, with full adjustment for clinical confounders, revealed six nominally significant differences in lipoprotein composition (all $p<0.05$), all in compositional fractions rather than absolute concentrations. Murter individuals showed higher proportional free cholesterol and cholesterol ester content within large and extra-large VLDL particles (L_VLDL_FC_pct, L_VLDL_C_pct, XL_VLDL_FC_pct, S_VLDL_CE_pct), alongside lower proportional phospholipid content in small HDL particles (S_HDL_PL_pct) and lower cholesterol ester proportion in IDL particles (IDL_CE_pct). Since both groups share the same diagnosis and treatment, these compositional differences are unlikely to reflect disease stage or therapy effects. Targeted interrogation of the 2,000 disease-segregating SVs identified a 72 bp heterozygous intronic insertion in *FADS2* (chr11:61824394; genotype 0/1; absent from CoLoRS), encoding fatty acid desaturase 2, the rate-limiting enzyme in the conversion of linoleic acid to long-chain polyunsaturated fatty acids, at an established HT-GWAS locus (1). However, the insertion does not overlap any annotated regulatory element, and no supporting eQTL or chromatin interaction data were identified.

Discussion

The family-specific metabolomic comparison revealed altered lipoprotein compositional fractions within VLDL and HDL particles relative to unrelated treated HT patients, a pattern consistent with differences in enzymatic lipoprotein remodelling. However, no disease-segregating SVs were identified in canonical lipoprotein remodelling genes (*CETP, LIPC,*

LCAT, PLTP), and the small cohort size (n=7) precludes definitive conclusions. A 72 bp intronic insertion in *FADS2* represents a point of genomic–metabolomic convergence at an established HT-GWAS locus, but in the absence of regulatory element overlap or functional evidence, it does not meet the interpretive standard applied to the other prioritised loci in this study. These metabolomic observations are therefore best interpreted as hypothesis-generating, warranting replication in independent family members or carriers of the identified SVs.

References

1. Teumer A, Chaker L, Groeneweg S, Li Y, Di Munno C, Barbieri C, et al. Genome-wide analyses identify a role for SLC17A4 and AADAT in thyroid hormone regulation. Nat Commun. 2018 Oct 26;9(1):4455. doi:10.1038/s41467-018-06356-1 PubMed PMID: 30367059.
